# Working Memory Cells' Behavior May Be Explained by Cross-Regional Networks with Synaptic Facilitation

**DOI:** 10.1371/journal.pone.0006399

**Published:** 2009-08-04

**Authors:** Sergio Verduzco-Flores, Mark Bodner, Bard Ermentrout, Joaquin M. Fuster, Yongdi Zhou

**Affiliations:** 1 University of Pittsburgh, Department of Mathematics, Pittsburgh, Pennsylvania, United States of America; 2 MIND Research Institute, Santa Ana, California, United States of America; 3 Johns Hopkins University, Department of Neurosurgery, Baltimore, Maryland, United States of America; 4 Semel Institute for Neuroscience and Human Behavior, University of California Los Angeles, Los Angeles, California, United States of America; Indiana University, United States of America

## Abstract

Neurons in the cortex exhibit a number of patterns that correlate with working memory. Specifically, averaged across trials of working memory tasks, neurons exhibit different firing rate patterns during the delay of those tasks. These patterns include: 1) persistent fixed-frequency elevated rates above baseline, 2) elevated rates that decay throughout the tasks memory period, 3) rates that accelerate throughout the delay, and 4) patterns of inhibited firing (below baseline) analogous to each of the preceding excitatory patterns. Persistent elevated rate patterns are believed to be the neural correlate of working memory retention and preparation for execution of behavioral/motor responses as required in working memory tasks. Models have proposed that such activity corresponds to stable attractors in cortical neural networks with fixed synaptic weights. However, the variability in patterned behavior and the firing statistics of real neurons across the entire range of those behaviors across and within trials of working memory tasks are typical not reproduced. Here we examine the effect of dynamic synapses and network architectures with multiple cortical areas on the states and dynamics of working memory networks. The analysis indicates that the multiple pattern types exhibited by cells in working memory networks are inherent in networks with dynamic synapses, and that the variability and firing statistics in such networks with distributed architectures agree with that observed in the cortex.

## Introduction

Persistent elevation in firing rates of cortical neurons during retention of memoranda has been suggested to represent the neuronal correlate of working memory [1]–[3]. This activity in so-called memory cells (as observed in microelectrode recordings of neurons in the cortex of primates during the performance of delay tasks) exhibits a number of different general patterns. One pattern consists of cells whose elevated firing rate persists, on average across trials of the delay task, at the same rate for the entire period during which information of the memorandum is maintained in working memory. This type of dynamics represents the canonical bistable activity which has been a major focus of theoretical and computation modeling. A second elevated firing rate pattern consists of cells whose rate either decreases or increases throughout the memory period of a delay task. In decreasing-rate memory cells, the elevated activity is attuned to the memorandum (cue) of the task, and firing rate decays as the delay progresses towards the response of the task. In increasing-rate or ramping cells, elevated activity is motor- or response-coupled, and firing rate accelerates as the response of a task approaches. These rate-changing pattern cells and their respective networks have been suggested to represent two mutually complementary and interactive representations engaged in the transfer of information of cross-temporal contingencies from memory to action in working memory. Cells exhibiting these pattern types have been found to occur anatomically intermixed in the cortex [4], with cue- and response-coupled cells appearing to be more common than fixed delay rate cells [5]. In addition to these persistently elevated firing rate patterns, neurons which presumably are constituent members of working memory networks exhibit analogous inhibited (below baseline rate) firing patterns. Finally, many cells exhibit firing rate changes correlated with different working memory task events such as the presentation of memoranda (cue period) and/or the response of the delay task, but maintain baseline firing rates throughout the delay period during which the memorandum is retained in active short-term memory.

While the mechanism(s) by which the patterns of activity are initiated and maintained in working memory are undetermined, a number of plausible hypothesis have been proposed. With respect to persistent elevated-rate patterns, prevailing ideas which have emerged from computational and theoretical studies are that the activity arises as stable states in recurrent attractor networks [6]–[16] and/or inherent cellular dynamics [17]–[24]. These studies have had success in reproducing general bistable memory behavior. For example with respect to network studies, successful working memory behavior has been attained as defined by achieving persistent increased firing rates of cue-specific subpopulations of units in networks during the putative memorandum retention period of simulating delay tasks. A difficulty typically encountered however, is obtaining memory behavior, as defined within the specific range of frequency rates, statistics, and with the variability as observed in real neuronal populations of cortical working memory networks across the range of different persistent patterned behaviors. Neurons exhibiting each of the different persistent activity pattern types with some overall average frequency do so only as an average across multiple trials of a working memory task. Individual cells exhibit a significant amount of variability however, both in terms of firing frequency within and between trials of working memory tasks and may even exhibit different patterned behaviors from trial to trial [5]. Thus while cells exhibit one of the given patterns described above with some overall average firing frequency across many trials (as observed for example in an average peristimulus time histogram), they exhibit different average firing rates and/or pattern behaviors from trial to trial of the working memory task.

A potential source of these and other difficulties [25], is that they are examined within the framework of static synaptic structures. Specifically, the networks have fixed architectures, and are trained such that the strength of the connections between units (the weight matrices) produce desired memory behavior. Once memory behavior is achieved, the weight matrix is held constant. However, the simplification of fixed synaptic strengths may not be physiologically reasonable in light of the highly dynamic structure of the cortex. Cortical networks, and their constituent neurons, receive constant input from both external and internal sources, with learning and plasticity occurring concurrently with behavior. From a functional standpoint, fixed connection strengths necessarily limits the number of activities a network can perform, which could be undesirable given the plasticity of cortical function. Further, the ubiquity of cortical working memory [26] suggests that its associated activity might not occur in fixed, dedicated networks, but rather may arise from processes present in networks performing a variety of different functions [27].

Functional architecture is a second consideration of potentially fundamental importance to the dynamics of working memory networks. Typically, efforts have focused on studying working memory within the framework of local modules or networks that exist at various specific or general locations in the cortex. However, while working memory and/or working memory-correlated neuronal activity may be maintainable within local networks (or even cellularly), considerable evidence from neurophysiological and imaging studies have shown that working memory involves widely distributed cortical networks across multiple cortical areas [27]. Such a widely distributed architecture, which, if not fundamentally necessary for producing the firing rate patterns observed in working memory network cells, is probably active in the modulation of that activity. This modulation might entail not only producing the specific range of firing rates, but also the range of pattern types.

Recent work has indicated that working memory networks incorporate dynamic synapses. One study [28] revealed that connections between pyramidal cells in the prefrontal cortex exhibit facilitation, while others have demonstrated that neocortical synapses undergo substantial synaptic plasticity following synaptic activity [29], [30]. Particularly, it has been found that cells in certain cortical regions exhibit increased responses to sequences of theta burst stimulation, both from burst to burst within a given burst sequence, as well as across successive sequences. Work by Hempel et al., [31], and Galaretta and Henstrin [32] indicated that cortical synapses can exhibit augmentation (from 15 to 60 percent) that correlates with the frequency and duration of tetanic stimulation–similar to that frequently observed during the presentation of memoranda in working memory tasks.

Several computational efforts have attempted to address various aspects of the issues described above. For example, one study demonstrated that persistent activation with realistic frequencies might be achieved if working memory corresponds to attractor states on the unstable branch, and have proposed mechanisms by which such states might be stabilized [33]. Other work has emphasized the potential role of dynamic synapses in working memory processes, examining the effects of dynamic synaptic augmentation and rapid Hebbian plasticity in a recurrent network framework [25]. This work indicated that synaptic augmentation can reduce the amount of prior structure required for persistent activation to take place, while rapid Hebbian plasticity could enable persistent activity to take place within firing rate ranges observed in real cortical neurons. More recent studies have demonstrated that combinations of synaptic depression and facilitation might extend the attractor neural network framework to represent time-dependent stimuli [34]. Further efforts have indicated that calcium media synaptic facilitation could produce bistable persistent activation with firing rate increases typically observed in real cortical cells [35].

While working memory models have mostly concentrated on bistable persistent activation, some efforts have also addressed the issue of cue- or response-coupled patterns of activity that steadily increase and decrease during delay periods. For example, graded activity in recurrent networks with slow synapses has been modeled [24], while another recent study examined such activity in uniform recurrent networks with stochastic bimodal neurons without NMDA-receptor-mediated slow recurrent synapses [36]. This work has indicated that graded memory activity could be very difficult to produce within a single population or local module. Still other studies have examined the ability of networks to produce ramping behavior by maximizing the time the systems trajectory spends around the saddle node of the system's phase space [37]. Other work, while not necessarily producing working memory cells with firing rate statistics of real cells, has examined networks that produce the types of general patterns observed in working memory [38], [39]. A distributed network architecture may be crucial in understanding and producing those patterns of activity.

In this work we examine a cortical model of working memory incorporating dynamic synapses both within a local and a distributed cortical framework. We investigate the mechanism of dynamic synaptic facilitation in the generation of all of the different patterns of persistent activity associated with working memory and the effect of a distributed cortical architecture on the dynamics of working memory patterns. We first examine a firing rate model incorporating dynamic synapses representing a working memory network residing locally in a given cortical area. We analyze the statistics and firing-rate-patterns of this network during simulated working memory and compare the results with that of real cortical neurons recorded from parietal and prefrontal cortex of monkeys performing working memory tasks. A reduction of this model to a 2-dimensional system enables an analysis to completely characterize the states of the system. We then examined a distributed firing rate model consisting of 2 and 4 locally interconnected networks, analyzing the possible states as a function of different long-range connectivity schemes and strengths. The expansion of the architecture to multiple networks allows the incorporation of possible heterogeneity. We compare the output of these models (local and global architecture) with the activity of the database of real cortical neurons recorded extracellularly from the prefrontal and parietal cortex of primates performing working memory tasks. The model expands on previous work examining the ability of population models with dynamic synapses to produce bistable memory states, or rate changing states (either cue dependent during the stimulus period—i.e. Barak and Tsodyks [34]—or exclusively rate changing during the delay (Durstewitz [37]) to produce all different patterns (including inhibitory patterns) recorded during the delay period, and that these patterns can change their temporal features to accommodate a continuum of delay periods, as well as possessing relative rate changes and statistics as recorded in real cortical neurons. We also demonstrate that different patterns can occur in a distributed network concomitantly in a complimentary fashion as observed in the cortex. From the mean field firing rate model, a spiking network model is obtained whose population's mean firing rate corresponds to that of the firing rate model. This enables direct comparison of the activity with real cortical neurons. We examined the effect on unit activity with this spiking network with a distributed architecture consisting of up to four local networks connected by long range projections. The patterns and statistics of these spiking networks are analyzed and directly compared with the range of activities and firing statistics observed in the database of real cortical neurons. Finally we quantify the variability in spiking unit activity as observed in real cortical networks, and demonstrate from a nonlinear analysis how this activity arises. The results are compared to that observed in the real cortical cell populations. The results of this work demonstrates that all of the firing patterns correlated with working memory are inherently generated in distributed networks incorporating dynamic synapses, and these exhibit variability and firing rate statistics in agreement with what is observed in the cortex.

## Methods

**Figure 1:**
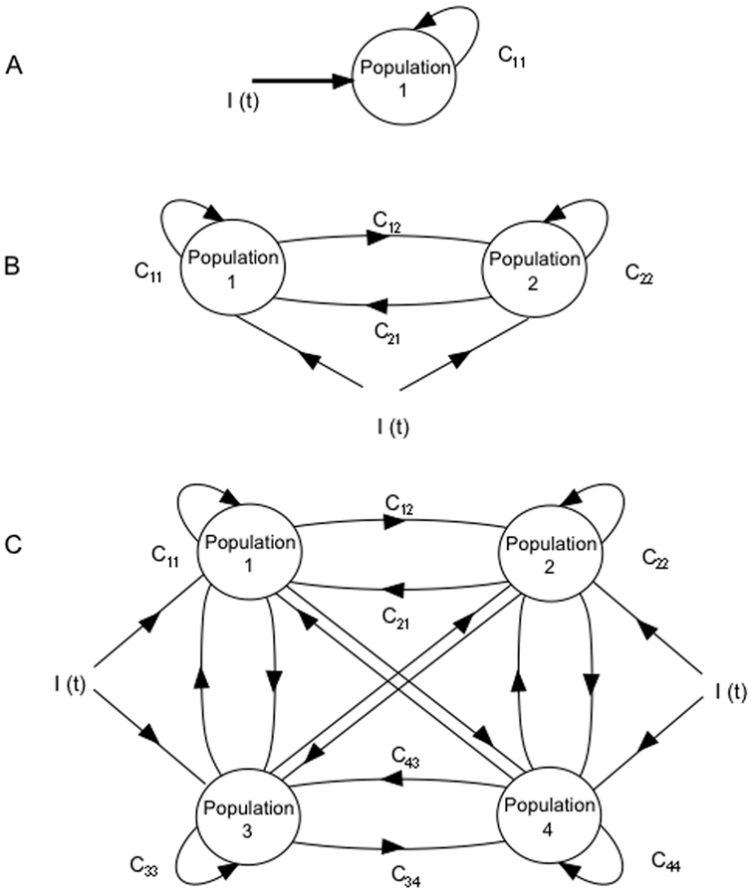
Schematic diagrams of network architecture of the single population, two population, and four population models. A) Single-population firing rate model. In the single-population model the network receives input from an external current *I(t)* during the cue period of a simulated working memory task. The synaptic activity *S(t)*, and consequently the firing rate increases from the input of the external current, resulting in a dynamic increase in its effective self connectivity *C_11_* as a result of a concomitant dynamic change in synaptic facilitation *W_1_*. B) 2-population distributed model. In the distributed 2-population model, 2 “local” networks, 1 and 2 are connected recurrently by long-range projections *C_12_* and *C_21_* whose strength is weaker than the populations' self connectivity *C_11_* and *C_22_*. Both populations receive input from an external current *I(t)* during the cue period of simulated working memory task. This results in changes in their respective population firing frequencies along with changes in their effective long-range projections and self couplings through dynamic facilitation. In the spiking unit version of the model, populations 1 and 2 consist of networks of 100 (or 1000) spiking units each with all-to-all connectivity. C) 4-population model. In the 4-population model, four local networks (1, 2, 3, and 4) all receive input from an external current *I(t)* during the cue period of the simulated working memory task. Each local population receives input from self connectivity, and weaker input from the long-range projections from the other populations. Long-range projections between the population pair 1 and 2, and between the pair 3 and 4 (i.e. *C_12_, C_21_, C_34_,* and *C_43_*) are stronger than the connection strength between populations 1 and 3 or 4, or between 2 and 3 or 4. In the spiking unit version of the model with 200 units, each population corresponds to a network of 50 spiking units with all to all connectivity. In the spiking unit version of the model with 2000 units, each population corresponds to a network of 500 units with all to all connectivity. The strength of the connections is scaled such that the total strength of connectivity to units is the same as the 200 unit network.

We start with a firing rate model of a local network (Figure 1A). While the population might correspond to a network anywhere in the cortex, for convenience for comparison with the real cortical data, we might associate it with a working memory network in prefrontal or parietal cortex. The network equation describing the synaptic activity of the population is given by
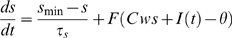
(1)where S denotes synaptic activity. The second term in (1) corresponds to the firing rate of the population with the function *F(X)* given by
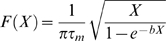
(2)in which *τ_m_* is the membrane time constant, and b is a parameter inversely proportional to the noise. This form of the firing rate function mimics the firing rate of a class I neuron in the presence of noise (∼1/b) [40]. The parameter *C* in equation (1) is the strength of feedback connections in the population,*τ_s_* is the decay constant for synaptic activity, *w* corresponds to the synaptic facilitation, and *θ* is the threshold. *I(t)* corresponds to an external current which increases during memorandum (cue) presentation in the simulated working memory task.

Dynamic synaptic facilitation (*w)* is incorporated in the model according to 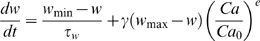
(3)where *τ_w_* is the decay constant, γ is a proportionality constant controlling the amount of facilitation as a function of intra-cellular calcium, and *Ca* is the calcium concentration. *Ca_o_* is a reference parameter controlling the level of intracellular calcium at which facilitation begins to increase. The calcium concentration dynamics are given by 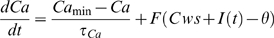
(4)where *τ_ca_* is the decay constant, and *F(x)* is of the form given in equation (2).

The above local architecture of the model is expanded to a distributed one, first through the addition of a second population, coupled to the first by recurrent long-range projections (Figure 1B). This allows the introduction of heterogeneity into the network as well as representing the first step towards investigating the effect of a distributed architecture on the dynamics and states of working memory. The system dynamics are described by the coupled network equations describing the synaptic activity
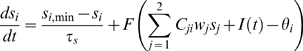
(5)where i = 1, 2 corresponding to the two populations.

The two populations can be considered to reside in different cortical areas (i.e. prefrontal and parietal cortex) or two populations within the same area. For convenience of description we can consider the populations to represent networks in different cortical areas, which for purposes of association with the real cortical data we take as prefrontal cortex (population 1) and parietal cortex (population 2). In these equations then, *C_12_* represents the strength of the projections from parietal cortex to the prefrontal cortex population, and *C_21_* is the connection strength from the prefrontal population to the parietal population, while *C_11_* and *C_22_* are the connections strengths within the prefrontal and parietal populations respectively. Synaptic facilitation is given by equation (2) and Calcium dynamics satisfy equations similar to (3) which are:
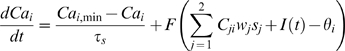
(6)


The distributed architecture is further extended to one consisting of four populations (Figure 1C), by recurrently connecting two of the 2-population models above such that every population has projections to every other population. The system dynamics are given by: 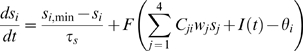
(7)where i = 1, 2, 3, 4 corresponding to the 4 populations, and with analogous extensions of equations (6) controlling the calcium dynamics. In this network each pair of populations (i.e. populations 1 and 2, and populations 3 and 4) are more strongly coupled to each other than they are to populations of the other pair. The network can be considered now to represent two local networks consisting of 2 populations each, residing within different cortical areas (i.e. prefrontal and parietal cortex). Thus the effects of heterogeneity may be examined, in addition to the effect of a distributed architecture on working memory dynamics and states. Particularly it allows the examination of the effect of heterogeneity and a distributed architecture on the occurrence of “complementary” working memory behaviors indicated by experiments to be simultaneously present in networks in the cortex.

**Figure 2:**
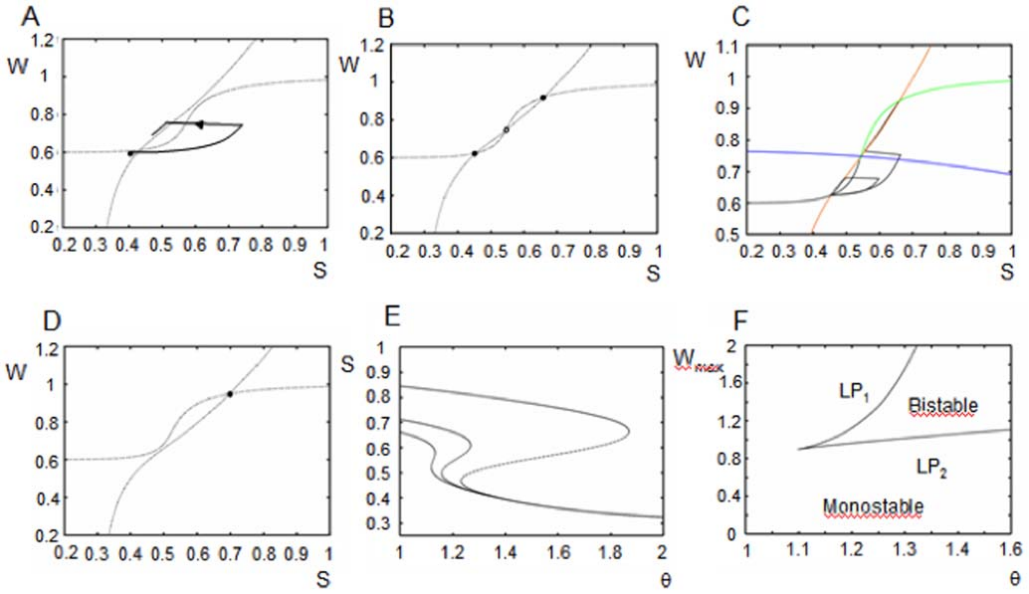
Phase portraits and bifurcation diagrams of the single population reduced model obtained for different values of the self connectivity *C_11_* and the maximum facilitation *W_max_*. The reduced 2-dimensional model is obtained at steady state calcium concentration (*dCa/dt* = 0). Shown in the phaseplane are the nullclines for Facilitation (*W*) and Synaptic activity (*S*). A) Nullclines with self connectivity *C_11_* = 4.8. The stable nodes of the system are where the nullclines cross. Here there is a single fixed point (solid black dot) corresponding to the baseline state. The trajectory of the system during a working memory task is indicated by the black line with an arrow. During the sample period, the applied current *I(t)* raises the synaptic activity, and firing rate, with a concomitant rise in the facilitation (approximately 33% increase). After a 300 ms cue period, *I(t)* becomes 0, and synaptic activity rapidly decreases towards the facilitation (*W*) nullcline, and the bottleneck. Because of the bottleneck, the trajectory then returns very slowly along the path of the facilitation nullcline towards the stable baseline state. In the present example, the synaptic activity and consequently the firing rate is still above the stable baseline rate at the end of the 10-second delay period, as indicated by the termination of the trajectory line. The system therefore maintains an increase from its baseline firing rate for the duration of the delay period. A continuum of elevated delay rates occur for different values of the parameters. B) *W* and *S* nullclines of the single population network with *C_11_* = 5. Note there are now 3 stable points where the nullclines cross: 2 attracting (black dots) and one saddle node (white dot). C) Possible system trajectories of the network in (B) with *C_11_* = 5. The saddle separatrix is indicated by the blue line. Two different possible trajectories of the system are shown which result from varying the magnitude of the external current *I(t).* For *I(t)* = 0.5 the trajectory does not cross the saddle separatrix and stays in the basin of attraction of the baseline state. In this case, given sufficient time the system returns to the baseline stable state. The resulting pattern of behavior is that of cue-coupled or decaying memory cells. For *I(t)* = 0.75 the trajectory cross the saddle separatrix, entering the baseline of attraction of the higher firing rate stable state. The system for this trajectory is shown to be in the higher firing rate state by the end of the delay period. The resulting pattern of behavior is that of response-coupled or ramping cells. In both cases the bottleneck can be adjusted such that the rate at which the system returns to the baseline state, or approaches the higher firing state can be arbitrarily slow, resulting in a continuum of different average firing frequencies during the delay, and apparent bistability at frequencies between the two stable fixed points. D) Phase portrait with *C_11_* = 5.2. The system again possesses a single stable state at a higher rate than in (A). E) Bifurcation diagram of steady state synaptic activity as a function of maximum facilitation (*W_max_*). Curves shown are for *W_max_* equal to 0.925, 1, and 1.25 (producing synaptic facilitation in the 30–60% range). Solid lines indicate stable fixed points, hashed lines are unstable fixed points. Note that 3 fixed points are present over a wide range of the facilitation. F) Bifurcation diagram for the parameters of maximum facilitation W_max_ and the threshold *θ*. The curves correspond to the limit points at different values of these parameters. The two branches of the limit points meet at a cusp point. For the region interior to the two curves there are three rest states and bistability, and outside them there is a single rest state and monostability.

We begin the analysis first from the single population model. The single population possesses 3-dimensional dynamics in the variables for synaptic activity (*S*), facilitation (*W*), and calcium concentration (*Ca*). A reduction of this model to 2 dimensions is achieved by assuming steady state calcium (*dCa/dt* = 0) allowing the system to be rigorously analyzed. While assuming steady state calcium does not have an immediate justification from a neurophysiological standpoint, it produces a system with the same attractor structure as the 3-dimensional system and thus allows the rigorous analysis. We carried out analysis of the dynamics and the stability of states of the model using XPPAUT [41]. For the 2-dimensional reduced model we examined the phase portraits (Figure 2), from which the fixed points of the system and their stability were determined. Through this analysis, a range of biologically plausible parameters were determined which generate persistent working memory pattern types with statistics in the range typical of real cortical neurons (Table 1). These network parameters were then used in the full 3-dimensional model with dynamic calcium. For the 3-dimensional model, the fixed points of the system were first examined to determine coincidence with the 2-D model. Simulated working memory tasks were then run, varying the magnitude of the facilitation, self connectivity, and the magnitude of the input current. The firing rate patterns and frequencies exhibited by the model were compared to the types of patterns and frequencies observed in the database of parietal and prefrontal neurons recorded from monkeys during performance of working memory tasks in other studies. [42]–[44]. A simulated trial of a working memory task followed approximately the same generic sequence as that for which the single neuron database was acquired. The simulated task consisted of a 20-second baseline period (during which the population was in a baseline firing fixed point), followed by a 300-ms sample cue period corresponding to the period during which a memorandum was presented. After the cue period, a 12 second delay period followed. We did not consider in this study a behavior/motor response period following the delay, but rather restricted our analysis to the network behavior during these first 3 temporal aspects of the working memory task. To analyze the firing rate patterns and firing rate statistics of the model, peristimulus time (PSTH) histograms were generated and analyzed over a range of values of the self connectivity and facilitation. In addition, the phase diagram of different possible pattern states occurring over a range of values of the self connectivity and maximum synaptic facilitation were examined. For the single population model, PSTH histograms and phase diagrams were also generated for different values of dynamic synaptic depression and self connectivity, and the resulting patterns and firing rates were analyzed. Synaptic depression was incorporated by allowing the parameter for maximum facilitation (*Wmax*) to range over values less than the value of the baseline facilitation (*Wmin*) in equation (3).

**Table 1:** Network parameters.

Mean wmax	1
Std. Dev. wmax	0.02
wmin	0.6
γ	8
τ_w_	2
τ_s_	0.05
τ_Ca_	0.5
β	0.5
s_min_	0.3
E	6
Ca_0_	82
Ca_min_	8
amp	0.2
θ	1.2
τ_m_	0.03

Model parameter values obtained from analysis of the reduced 2-dimensional network. These parameters are used in all of the local and distributed networks.

For the distributed 2-population model, the parameters used were within the ranges determined and used in the single population model, and simulated working memory trials were conducted following the same course as that used for the single population model. Firing rate patterns and statistics of both populations were analyzed over a range of the inter-population connectivity values. PSTH histograms were generated to analyze the firing rate patterns and statistics. Phase diagrams of the firing rate patterns occurring in each population were generated as a function of the inter-population connectivity strength. An analysis of the behavior of the entire network was carried out through an examination of the possible pattern types occurring concomitantly in the two populations. This analysis was carried out by examining overlapping patterns in the phase diagrams of the two populations.

For the distributed 4-population model, the parameters for each population were within small ranges of those determined and used in the preceding single- and 2-population models. Simulated working memory trials were conducted following the same previous course as well. Firing rate patterns and statistics occurring in all four populations were analyzed and compared to the activity of the real parietal and prefrontal neurons. Phase diagrams were generated of the different firing rate patterns occurring in the populations as a function of different inter-population connectivities. An analysis of the behavior of the entire network was carried out through an examination of the possible different pattern types occurring concomitantly in the different populations. This analysis was carried out through an examination of overlapping states in the phase diagrams of the four populations. Resulting behaviors were compared with that of the 2-population model.

Having determined the dynamics through the study of the firing rate models, spiking models were generated to make direct comparison with the single unit data. Spiking model versions of the 2- and 4-population firing rate models were generated by replacing the populations' activities first with networks of 200 spiking units exhibiting the same overall mean firing rates. The network consisted of spiking neurons with all-to-all connectivity and random strengths. Connections between populations were both excitatory and inhibitory. Specifically, mean connectivity values were chosen from regions of the phase diagrams of the mean field model in which the range of memory cell pattern types were robustly exhibited. These connectivity values were then used as the values for setting the mean of the connectivity in the spiking model. Distributed spiking networks consisting of two populations of 100 units each, and four populations of 50 units each were generated with the average inter-area connectivity chosen to match the mean field model values within ranges of the standard deviation (Tables 2 and 3).

**Table 2:** Mean connectivity values for the 2 population network.

	from A	from B
to A	0.05 (0.01)	−0.00134 (0.001)
to B	−0.0145 (0.04)	−0.057 (0.005)

The mean values of connection strength between populations in the 2-population models. These values were used in the firing rate model, and also represent the mean connectivity strengths between units of different populations in the spiking model. The standard deviations for each of the mean connection strengths are given in parenthesis.

**Table 3:** Mean connectivity values for the 4 population network.

	from A	from B	from C	from D
to A	0.101 (0.004)	−0.0012 (0.003)	−0.0014 (0.001)	0.0005 (0.001)
to B	−0.0462 (0.003)	0.1405 (0.003)	0.0011 (0.0001)	0.0002 (0.003)
to C	−0.0003 (0.003)	−0.0001 (0.003)	0.1025 (0.004)	−0.0034 (0.004)
to D	0.0003 (0.003)	0 (0.001)	−0.036 (0.003)	0.132 (0.003)

The mean values of connection strength between populations in the 2-population models. These values were used in the firing rate model, and also represent the mean connectivity strengths between units of different populations in the spiking model. The standard deviations for each of the mean connection strengths are given in parenthesis.

The spiking activity of the single units was modeled as theta neurons [40], [45]. Unit firing frequency as a function of the injected current (F-I curve) can be obtained analytically in the theta model. This F-I curve is a square root function which provides a correspondence between the firing rate model and the theta model. The F-I curve for the theta model is described by 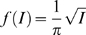



Whereas the curve for the mean field firing rate model is 
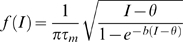



thus obtaining the correspondence between the mean field and spiking models (as the parameter *b* goes to infinity the above expression becomes the equation for a noisy integrate and fire model).

The membrane potential dynamics of a unit in the spiking model is given by the equation 
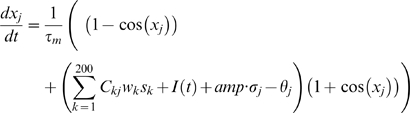
(10)where *I(t)* is an external current occurring during the presentation of memoranda, and *amp* is the amplitude of the Weiner noise. The synaptic activity of a unit *s_j_* in equation (10) increases with each afferent spike according to 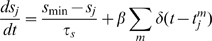
(11)where *β* corresponds to the increase in synaptic activity from a single afferent spike, *t^m^_j_* is the time of incidence of the m^th^ afferent spike on the j^th^ neuron, and *τ*_s_ is the decay constant. The dynamics of the synaptic facilitation *w_j_* in equation (10) is given by 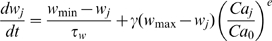
(12)where *Ca* corresponds to the intercellular calcium concentration which modulates the change in facilitation and increases with each spike according to 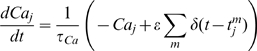
(13)


The 2-population spiking network consisted of two “local” networks of 100 neurons each with all-to-all connectivity, and with average weaker recurrent connectivity between populations than within the populations. The activation properties of each individual network reflect that of the single populations of the firing rate models.

For the 2- and 4-population spiking networks, working memory task simulations were conducted similarly to those for the firing rate model, and the firing rate patterns and statistics were analyzed. During the baseline period of the simulated working memory task, facilitation in the models was kept low such that the firing rate of the populations was near the baseline fixed-point attractor state inherent in the model (as determined from the phaseplane analysis of the firing rate model). After 20 seconds, the baseline period ended and an external current *I(t)* was applied for 300 ms. The external current raises the firing rate of many units in the populations, simulating the activity observed during presentation of the memorandum in working memory tasks. The current input and increased firing rate triggers dynamic facilitation through equations (11–13). After the cue period, the delay period begins. For the spiking model simulations, unit activity was analyzed over an 11-second delay period which is proportional to the delay period of the working memory tasks during which the parietal and prefrontal cells of the database were recorded. PSTH histograms of units were generated to analyze the patterns and firing rate statistics of the units. Average PSTH histograms were generated for each unit over 10 simulated working memory task trials. Pattern types appearing in the average PSTH histograms were determined and the distribution of patterns in the network were compared to the distribution of patterns observed in the parietal and prefrontal neuron populations of the database. Variability in working memory patterns occurring across trials for each unit was analyzed and compared between the 2- and 4-population networks and the neuronal populations.

To examine the effect of network size on patterns exhibited in the networks across trials and their variability, we generated 2- and 4-population networks consisting of 2000 spiking units. For these networks the distribution of pattern types exhibited on each of 20 simulated working memory task trials was obtained and the average distribution across all 20 trials was determined. These distributions were compared to the distributions obtained with the 200 unit networks as well as that observed in the parietal and prefrontal neuron populations of the database. Variability in firing rate within trials was determined through an analysis of the coefficient of variation (CV) of the ISI's during the baseline and delay periods. Variability in working memory patterns occurring across trials for each unit was analyzed and compared to that observed in the 2- and 4-population networks of 200 units.

The database with which the different models' activity is compared consists of 812 neurons recorded extracellularly from the parietal cortex (Brodmann areas 2, 3, 5, 7) and prefrontal cortex (areas 6, 8, 9 and 46) of monkeys performing working memory tasks. In parietal cortex, 521 cells were recorded from monkeys during performance of a haptic delayed matching-to-sample task [42], and in prefrontal cortex, 291 neurons were recorded from monkeys during the performance of a cross-modal audiovisual delayed-response task [43]–[44]. The analysis of this database and the compilation of its statistics in terms of firing rates, patterns and statistics have been presented elsewhere [5].

## Results

### Single Population Model

The reduced single-population model is 2-dimensional (in the variables *S* and *W* for synaptic activity and facilitation respectively) and thus phaseplane and rigorous mathematical analysis was carried out. The nullclines of the system (Figure 2) correspond to the curves along which the synaptic activity and facilitation are constant (*ds/dt* = *dw/dt* = 0). The steady states of the system are defined by the points at which these 2 curves intersect. For sufficiently low self connectivity strengths (or low maximum facilitation), only one such point is present, corresponding to the baseline firing rate of the population (Figure 2a). Stability analysis reveals this is an attracting fixed point. Thus transient perturbations from the external current during the sample period (resulting in increased synaptic activity, firing rates and facilitation) ultimately relax back to this state. As the self connection strength (or amount of facilitation for a given input current) is increased, the *W*-nullcline intersects the *S*-nullcline at 3 points (Figure 2b). Stability analysis reveals that 2 of these nodes are attracting fixed points, and one is a saddle node. The presence of a stable state corresponding to baseline, and a second stable state corresponding to an above baseline firing rate, enables bistable behavior, although the difference in firing rates associated with these states is much larger than typically observed in cortical data over much of the parameter space. For example, in the subpopulation of memory cells recorded from the parietal cortex, 90.1% of the cells exhibited increases from baseline to delay of less than 10 Hz, and 69.9% of frequency changes were less than 5 Hz. In the subpopulation of memory cells recorded from prefrontal cortex 100% exhibited increases of less than 10 Hz, and 95.2% were less than 4 Hz. Persistent elevated firing rates within these ranges typically observed in cortical data is inherently prevalent in the model without incorporating many of the previous mechanisms providing solutions for acquiring that behavior (see for example Latham and Nirenberg, [33]; Barak and Tsodyks [34]; Mongillo et al., [35]). An essential feature of the model allowing this behavior is the presence of a bottleneck which appears in the phaseplane near the *S* and *W* nullclines corresponding to regions of greatly diminished rates of change for the dynamic variables. Further, a bottleneck is present over a broad range of the parameter space, and its presence is not dependent on fine tuning of parameters. The bottleneck comes about because the equations of the system are a continuous map approaching zero when the nullclines are close to each other in phase space. Thus as shown in Figure 2, the values of ds/dt and dw/dt are reduced in those areas. There are two factors in the decay rate which include the bottleneck and the value of the time constants. While the presence of a bottleneck is not a result of the difference in time constants (but rather the shape of the nullclines), in the present system the shape of the nullclines has a dependence on the value of the time constants, and thus the slower rate change in w than s contributes both in terms of the bottleneck's existence via nullcline shape, as well as acting to slow decay of its own accord. Thus both contribute to the slower decay. Because of the bottleneck however, the “effective time constant” or rate of decay is much slower than would be predicted from the actual time constants. When the firing rate is elevated above baseline by an external current during the cue period, facilitation also increases. The trajectory in the phaseplane is such that passing through the bottleneck, the return of the system to the baseline stable state (or procession to the higher firing rate attractor state) is “impeded”. Thus while not in a stable state, the system remains in a state of elevated (above baseline) firing frequency for an extended period of time, which can be virtually indefinite. Over a wide range of values of the parameter space, the decay to one of the stable states of the system is sufficiently slow such that no significant change in elevated firing rate is observed for the duration of the putative memory period. From the frame of reference of the memory task, this activity appears as bistable. In contrast to actual bistability of the model however, the difference in firing rates between baseline and delay periods for this apparent bistability can adopt a continuum of values within the range typically observed in real cortical cells (i.e. differences between baseline and delay rate less than 100% of baseline or typically<5 Hz). This is also true of both the decaying memory cell and ramping cell behavior. The range of facilitation values over which the different firing rate behaviors occur is affected by the value of maximum facilitation parameter. This parameter (and the threshold *θ*) significantly determines the fixed points of the system (where the facilitation nullcline intersects the synaptic activity nullcline). While this parameter is varied over a large percentile range (i.e. several hundred percent), the resulting change in actual facilitation realized by the network is within the range of 10% to 60%–within the range of reported increases (for example see Hempel et al. [31]).

We next analyze the stability of the attractor states of the network as a function of the threshold parameter (Figure 2E and 2F). The bifurcation diagram reveals that the 3 fixed points of the system (two stable nodes and one saddle point) are present over a wide range of this parameter. For parameter values where the trajectory remains below the stable manifold (within the basis of attraction of the baseline node), the system ultimately returns to that attractor state. For parameter values in which the trajectory travels above the stable manifold (into the basin of attraction of the stable node corresponding to the higher firing rate), the system approaches that second stable state.

**Figure 3:**
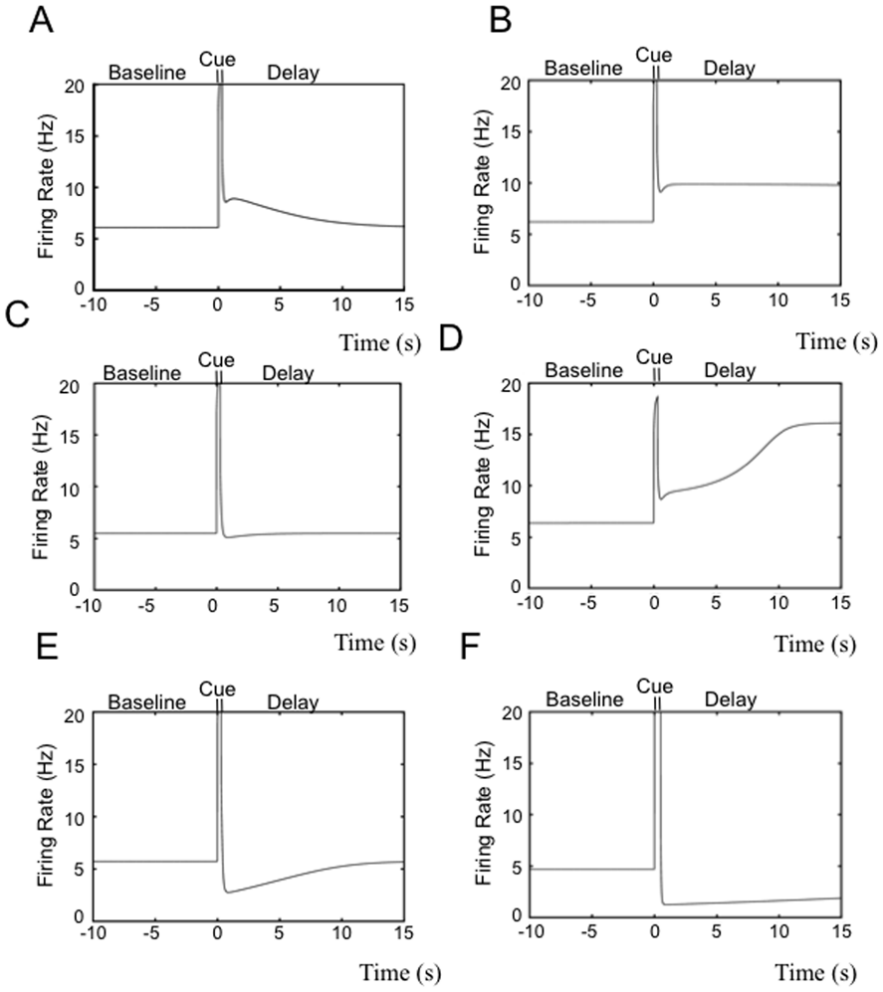
PSTH histograms of the single population firing rate model during the simulated working memory task. Different patterns correlated with working memory are obtained for different values of the facilitation (or depression) and the magnitude of the input current during the cue period. A) Decaying memory cell behavior. The baseline frequency is approximately 6 Hz, and at the beginning of the delay period the firing rate is approximately 9 Hz. The firing rate has returned to the baseline level by the end of the delay period. Average increase in firing rate from baseline to delay period is approximately 1.5 Hz. B) Apparent bistable memory cell behavior. The delay firing rate does not correspond to a stable state, but the decay towards the baseline state is sufficiently slow so that no significant decrease in firing rate occurs by the end of the period. The baseline firing rate is approximately 6 Hz, and the delay period firing rate is approximately 10 Hz. C) Nonresponsive pattern behavior. The population responds during the presentation of the cue, but immediately returns to and maintains the baseline firing rate during the delay. D) Ramping response-coupled cell behavior. The baseline frequency is approximately 6 Hz, and at the beginning of the delay period the firing rate is approximately 9 Hz. The firing rate adopts the rate of the higher fixed point attractor by the end of the delay period. Average increase in firing rate from baseline to delay period is approximately 6.5 Hz. E) Decaying inhibited pattern. In this example the value of maximum facilitation is less than the background facilitation, resulting in dynamic synaptic depression. The baseline frequency is approximately 6 Hz, and at the beginning of the delay period the firing rate has decreased to approximately 2.5 Hz. The firing rate has returned to the baseline rate by the end of the delay period, mirroring the decaying memory cell activity observed in (A). Average decrease in firing rate from baseline to delay period is approximately 1.75 Hz. F) Apparent stable inhibition. The delay firing rate does not correspond to a stable state, but the decay towards the baseline state is sufficiently slow so that no significant decrease in firing rate occurs by the end of the period. The baseline rate is approximately 4.5 Hz, and the delay period firing rate is approximately 1 Hz. Every pattern observed in the cortical database as reported in [5] is observed with the exception of delay inhibition that increases throughout the delay period.

Having determined the states of the 2-dimensional system, we next use the parameters of this 2-dimensional network (Table 1) in the 3-dimensional model with dynamic calcium. An analysis of the attractor states reveals that the 3-dimensional system retains the same attractors as the 2-dimensional system. Figure 3 shows PSTH histograms of the model during the simulated working memory task. These histograms show that the pattern activities observed in the trajectories of the phase portraits of the 2-dimensional model are present. Particularly this analysis shows that the network inherently exhibits the range of excitatory and inhibitory patterns correlated with working memory for different values of facilitation and self-connectivity. For values of facilitation (or input strength and/or duration) such that the trajectory of the system stays below the separatrix in the basin of attraction of the baseline attractor state, the population can exhibit persistent activity which decays towards baseline throughout the delay (Figure 3a). The achievable increases in firing rate from baseline to delay can take on low values (i.e.<5 Hz) and can adopt any rate within a continuum. The rate of decay of the persistent activation towards baseline is also variable along a continuum. For a range of trajectories and parameter values, the decay in firing rate during the delay can become slower and slower, to the point that the population approaches for all intents and purposes bistable behavior (Figure 3b). Once again, the increase in firing frequency during the delay can occur along a continuum. For sufficiently low values of facilitation, the population exhibits a non-responsive pattern (Figure 3C). That is the trajectory returns to baseline firing rates immediately following the cue. Thus the population responds to working memory events (i.e. the cue), but exhibits baseline rates throughout the delay. As facilitation increases (or the input strength/duration increases) such that the trajectory proceeds beyond the separatrix, the pattern becomes that of a ramping increase of firing rate during the delay period (Figure 3D). For sufficiently large values of facilitation, the system quickly adopts the higher firing rate attractor state, thus exhibiting bistability (with differences between baseline and delay period >10 Hz for most parameter values). Dynamic synaptic depression can be introduced into the network rather than facilitation for values of maximum facilitation (*Wmax*) that are less the background value (*Wmin*) in equation (3). For sufficiently small values of synaptic depression, as was the case for facilitation, the network exhibits the non-responsive pattern. As synaptic depression is increased the network exhibits the inhibitory pattern which is the mirror image of decaying memory cells (Figure 3E). As was the case for the excitatory memory cells, the decay back to the baseline state of the inhibitory pattern can be slowed to the point where the population exhibits an apparent fixed rate inhibition pattern (Figure 3F).

**Figure 4:**
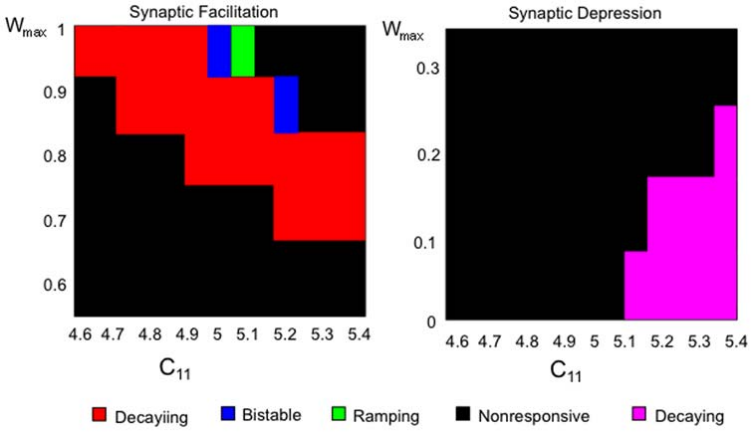
Phase Diagram of different patterns exhibited by the single population model as a function of the maximum facilitation parameter and self-connectivity. *Left*: Patterns exhibited with dynamic synaptic facilitation. Actual facilitation is less than the maximum facilitation parameter and falls within a physiological reasonable range (10–60%). *Right*: Patterns exhibited with dynamic synaptic depression which takes place when values of *W_max_* are less than the baseline level (*W_min_*). Pattern activity was determined by examining the firing rate activity during 3 periods of the working memory task: the first 5 seconds of the delay period (D1) immediately following the cue, the second 5 seconds of the delay period (D2), and the last 5 seconds of the baseline period (B) immediately preceding the cue period. Significant differences were taken to be present if the absolute difference between any two periods was greater or equal to 0.5 Hz, which is approximately the lower limit of significant differences observed in the real cortical parietal and prefrontal cells. Fixed rate memory cell behavior consisted of activity in which the absolute difference in average firing rate between D1 and D2 was less the 0.5 Hz, while both those periods exhibited an average firing rate greater or equal to 0.5 Hz above the baseline rate. Ramping cue-coupled cell behavior consisted of activity in which D2 exhibited an average firing rate greater or equal to 0.5 Hz above that of D1, and the firing rate of D1 was greater or equal to that of B. Decaying memory cell behavior consisted of activity in which D1 exhibited an average firing rate greater or equal to 0.5 Hz above both B and D1. Nonresponsive cell behavior consisted of activity in which the difference between any of the periods (B, D1, and D2) was less than 0.5 Hz. Decaying inhibition cell behavior consisted of activity in which the average firing rate during D1 was at least 0.5 Hz less than that exhibited during B and D2.

Figure 4 shows the phase diagram of the firing rate patterns of this model as a function of facilitation strength and self connectivity strength. It can be seen that the different excitatory patterns observed in the cortical data are produced (decaying memory, bistable memory, and ramping cells) over a wide range of parameters. As is the case in the cortical data, the non-responsive pattern behavior is the most prominent pattern type across the parameter space. For the case of synaptic depression, the decaying inhibition pattern occurs prominently over a range of parameters in addition to the non-responsive pattern.

The mechanism for the behaviors illustrated can be understood from the stability analysis and examination of the phaseplane (Figure 2). In all cases the firing rate begins at the lower attractor state with low values of facilitation. The input of current increases the synaptic activity *S*, and therefore firing rate and subsequently the facilitation *W* increases. If the self connectivity, facilitation or magnitude of the external current is sufficiently low such that the trajectory of the system does not cross the saddle separatrix, the trajectory is such that *S* quickly decreases until it approaches its nullcline. Here the trajectory proceeds such that it approaches the baseline stable attractor along the path of that nullcline. However, the bottleneck through which the trajectory proceeds slows the rate of return to the baseline state. The bottleneck can slow that rate such that the trajectory is impeded to the point that firing rate appears bistable with respect to the duration of the memory period of the task. As self- connection strength, synaptic facilitation or external current magnitude is raised beyond a critical point such that the system's trajectory goes beyond the saddle separatrix in the phaseplane, the system approaches the second stable state which corresponds to an above baseline firing rate—resulting in ramping or response-coupled cell pattern behavior. Once again the rate of this increase is affected by the bottleneck, and may be arbitrarily slowed such that the firing rate appears bistable with respect to duration of the memory period of the task. This phenomenon exists for a broad range of parameter values. Thus the inherent bistability in these cases is critical in modulating patterned memory behavior, but does not in many cases in and of itself represent the memory states. Rather the activation of the network itself could represent active working memory.

### 2-Population Firing Rate Model

**Figure 5:**
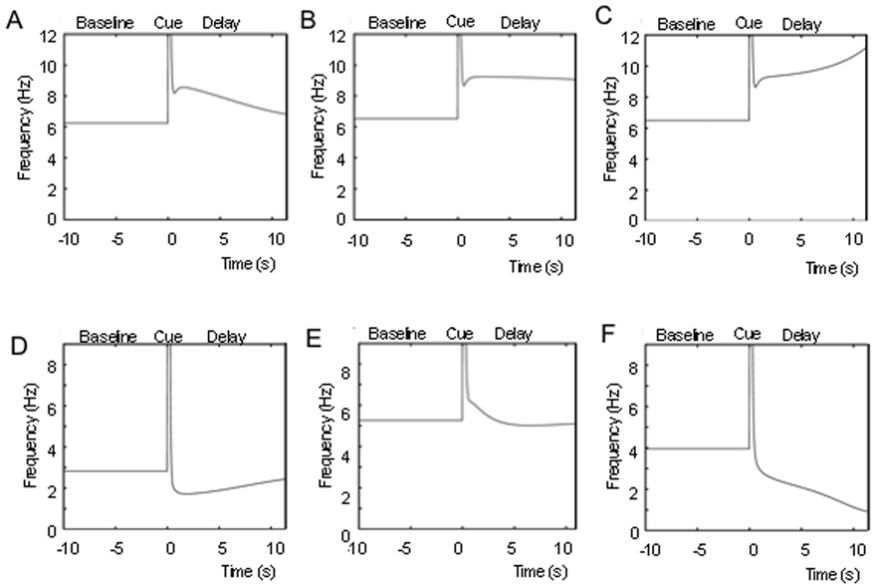
PSTH histograms of activity exhibited by the 2-population firing rate model at different values of the inter-area connectivity. All general patterns in the cortical data as reported in [5] are exhibited by the network, with frequencies in the range of the real data. Firing pattern behaviors shown are A) decaying memory cell, B) Stable memory cell, C) Set cell D) Decaying inhibition cell, E) Stable inhibition cell, and F) ramping inhibition cell.

**Figure 6:**
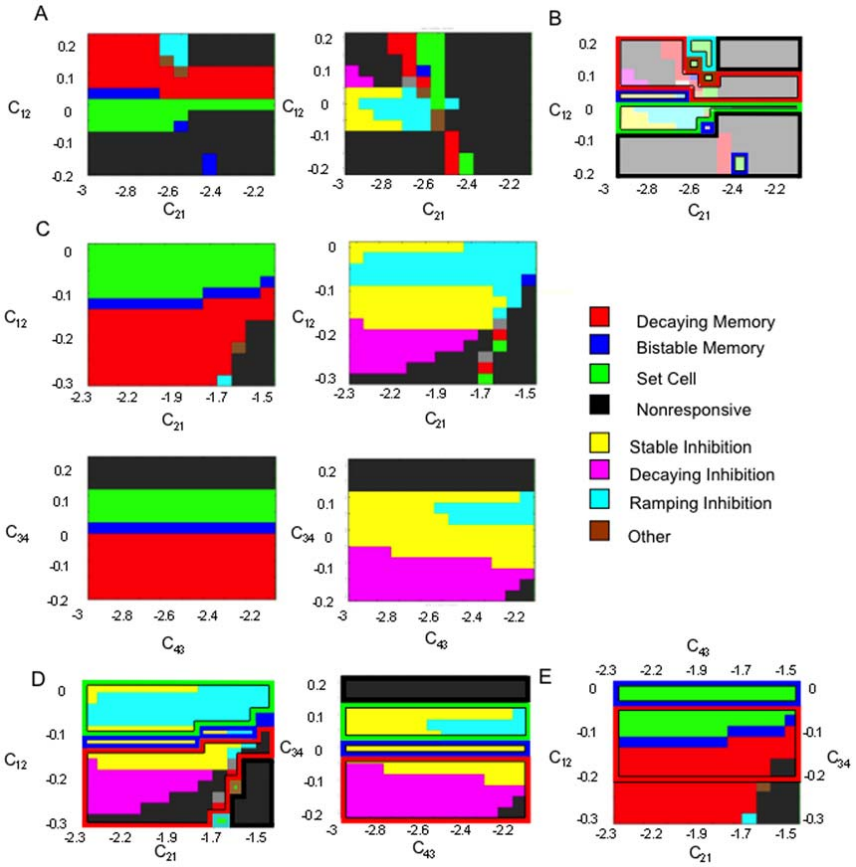
Phase diagrams of the patterns exhibited by the 2- and 4-population firing rate models. The phase diagrams were created using the same procedure carried out for the generation of the phase diagram of the single-population model. A) Phase diagrams of the activities of the 2-population model as a function of the inter-population connectivity. *Left*: Phase diagram of population 1. *Right*: Phase diagram of population 2. The self connectivity of population 2 (0.9) is slightly less than population 1 (1.0) so that the phase diagrams of the two populations are not identical. Excitatory-Inhibitory (E-I) and Inhibitory-Inhibitory (I-I) connectivity architectures between the populations are examined. The pattern category “other” corresponds to decaying or ramping cell behavior in which the average firing rate of one period of the delay–D1 or D2–is significantly less than the baseline (i.e. at least 0.5 Hz less than the firing rate during the period B), while the other is significantly greater (i.e. at least 0.5 Hz greater than the firing rate during the period B). Note that all of the pattern types (excitatory, inhibitory and nonresponsive) are obtained over a wide range of the parameters. B) The overlapping simultaneously occurring patterns in the 2 population network across the range of inter-population connectivity values. The figure shows the 1 and 2 populations' (left and right phase diagrams of panel A to the left) phase diagrams superimposed on each other. The 1-population states are shown as solid, and the corresponding 2-population states are shown in outline. Discrete regions of the overlapping phase diagrams reveal simultaneously occurring network behaviors observed in cortex during working memory. Included is simultaneous occurrence of decaying memory behavior in both populations (red-red overlap), and stable memory behavior in one population and ramping cell behavior in the other (blue-green overlap). Also common network behaviors observed are the simultaneous occurrence of the excitatory patterns in one population and the nonresponsive pattern in the other. Inhibitory patterns tend to occur in one population primarily with ramping cell behavior in the other. C) Phase diagrams of the activities of the 4-population model as a function of the inter-population connectivity. *Upper left*: Phase diagram of population 1. *Upper right*: Phase diagram of population 2. *Lower Right*: Phase diagram of population 3. *Lower Right*: Phase diagram of population 4. All the pattern types are exhibited by the network. However, the populations partition themselves such that they exhibit almost exclusively either excitatory or inhibitory memory patterns across the values of interpopulation connectivity. D) Simultaneously occurring patterns in populations 1 and 2, and in populations 3 and 4. E) Simultaneously occurring patterns in populations 1 and 3. The figure shows 1 and 3 (excitatory) populations' phase diagrams superimposed on each other. Over a significant range of the parameters, memory cell behavior and ramping cell behavior co-occur (red and green overlap). The existence of such simultaneous population behavior has been indicated in prefrontal cortex. Memory cell behavior is also seen to simultaneously occur in multiple populations across a wide continuous range of the parameter space (red-red and red-blue overlap).

We next analyze the behavior of a 2-population network, recurrently connecting two of the single populations. This model is 6-dimensional and thus cannot be easily reduced and analyzed as was the case for the single population model. We analyze the patterns and statistics through the PSTH histograms (Figure 5) and the phase diagrams (Figure 6) of pattern types as a function of the strength and sign (i.e. excitatory or inhibitory) for the net effect of the inter-population projections. The phase diagrams enabled the examination of possible concomitant activities in the different networks.

Inhibition, in addition to excitation, is incorporated in the mean field 2-population model via the inter-area projections between populations. While long-range projections in the cortex are excitatory, inhibition is examined as well according to the assumption that the majority of the long-range projections may project either to inhibitory or excitatory interneurons. Thus the net effect of these projections can be excitatory or inhibitory. We analyze the behavior of the network for different possible inter-population connectivity schemes (i.e. Excitatory-Inhibitory (E-I), and inhibitory-inhibitory (I-I). Slightly different values for self-feedback connections strengths within the two populations were chosen.

As was the case for the single-population model the PSTH histograms reveal that the 2-population model exhibits the excitatory patterns of memory and decaying-rate or ramping cells with a continuum of rate differences. The inclusion of inhibitory connections results in the presence of parameter ranges in which all of the inhibitory patterns (mirroring the excitatory ones) occur. These inhibitory patterns can occur purely as a function of inhibitory inter-population connectivity, without incorporating dynamic synaptic depression as was the case for the single population. In addition the inhibitory pattern of increasing inhibition throughout the delay (mirroring the excitatory ramping cells) which was absent in the single population model, now can occur (Figure 5f).

In the phase diagrams of the 2-populations (Figure 6A) it can be seen that all of the patterns of memory behavior occur over broad ranges of the parameters, and thus without fine tuning, in both populations. As in the single population model, the non-responsive type is the most prominently occurring pattern across the parameters, followed by decaying memory cells and ramping cells. Less commonly occurring types are fixed rate memory cells and the inhibitory mirror images of the excitatory patterns. While all the patterns occur over a broad range of parameters, the specific patterns present over given ranges varied considerably between populations. Thus many specific complementary patterned activities occurred simultaneously in both populations only over small ranges of the parameters, and thus some degree of fine tuning is necessary to achieve particular overall network behaviors. For example, as can be seen in the overlapping phase diagrams (Figure 6B), attaining memory cell behavior simultaneously in both cortical locations, or attaining complementary cue-coupled/response-coupled behavior, requires the connectivity of the network to be restricted to relatively small specific ranges of inter-population connectivity values.

### 4-Population Firing Rate Model

We next consider the effect on the states of the network when the model is extended to 4 populations. In the 4-population model all of the patterned activities continue to be present over a continuum range of increases and decreases in firing frequencies. However the distributed architecture results in a “specialization” of pattern activity within specific populations. As can be seen in the phase diagrams (Figures 6 C and 6D) each local network (populations 1–4) exhibits the non-responsive pattern and almost exclusively either the excitatory or inhibitory memory patterns across the range of connectivity strengths. A result of this specialization or partitioning of pattern types between the local networks is that, in contrast to the 2-population model, simultaneous complementary pattern behaviors occur far more robustly across wide parameter ranges. Thus for example attaining memory cell behavior simultaneously in multiple cortical areas, or attaining complementary cue-coupled/response-coupled patterned behavior does not require fine tuning to a small restricted range of connectivity values (Figure 6E).

### Spiking Unit Network: 2-Population Model

**Figure 7:**
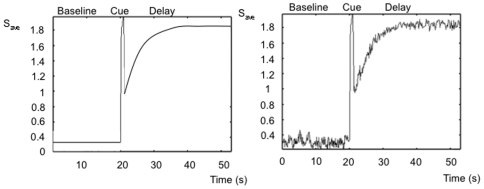
PSTH histogram of the single-population firing rate model (left) and the corresponding average spiking unit activity in a network of 50 units obtained from the spiking model (right). Note the general pattern is the same in both networks.

**Figure 8:**
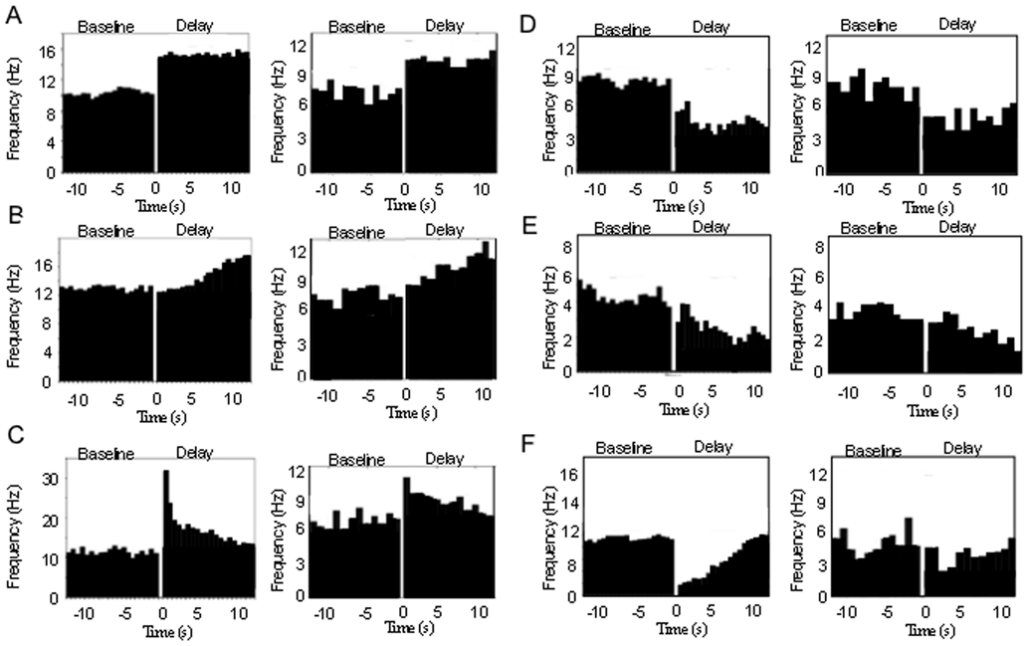
Average histograms from example units of the 2-population spiking model showing the excitatory and inhibitory working memory patterns, and average histograms from representative single neuron recordings from the database of prefrontal and parietal neurons exhibiting the same general patterns. PSTH histograms of spiking model units are shown in the left columns and real neuron histograms in the right columns. The gap between baseline and delay periods corresponds to the cue period (firing rate not shown). Histograms of the model units were averaged over 10 simulated trials of the working memory task. Patterns exhibited are A) Stable fixed rate memory (i.e. bistable) cell behavior, B) ramping cell behavior, C) decaying memory cell behavior, D) fixed rate inhibition cell behavior, E) ramping inhibition, and F) decaying inhibition.

**Figure 9:**
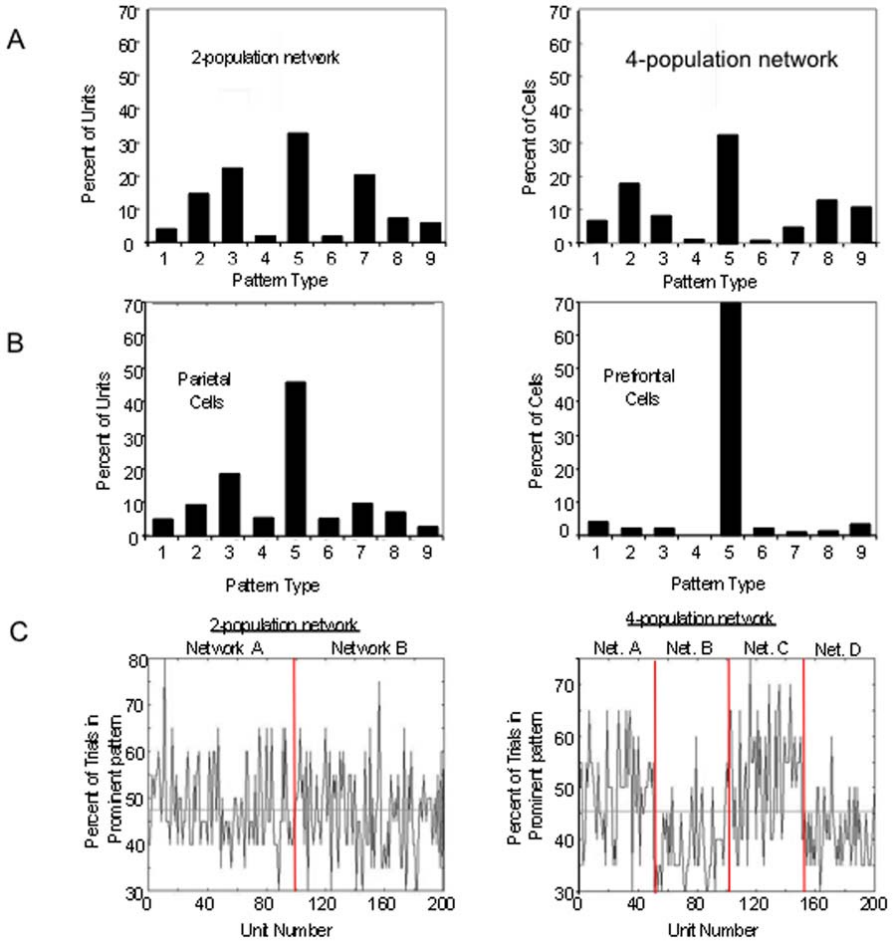
Distributions of units exhibiting the different pattern types in the spiking models and variability in pattern expressed from trial to trial in the units of those models. A) Distribution of pattern types exhibited by units in the 2- and 4-population spiking models (left and right respectively). Distributions correspond to the average number of units exhibiting each pattern type over 20 trials of the simulated working memory task. Pattern numbers correspond to the same patterns as labeled in table 4. Specifically 1) Ramping cells 2) fixed rate memory cells, 3) Decaying memory cells 4) ramping set cell with no frequency difference between D1 and baseline, 5) nonresponsive cell 6) ramping inhibited cell with no frequency difference between D1 and baseline, 7) decaying inhibition cell, 8) fixed rate delay inhibited cell, and 9) ramping delay inhibited cell B) Distribution of the percentage of parietal (left) and prefrontal (right) cells exhibiting each pattern type during the performance of unimodal and cross-modal working memory tasks. Note that the distribution of the real cells and the units in the spiking model are similar with nonresponsive cells being most common, excitatory memory cells (decaying, fixed rate, and ramping) being the next most common, and inhibitory patterns (decaying, fixed rate, and ramping) being the least common. Also patterns 4 and 6 (ramping excitatory and inhibitory cells with no frequency differences between D1 and baseline) occur infrequently in both the models and real cells. C) Consistency of pattern type expressed across trials for each of the 200 units in the 2-population spiking model (left) and the 4 population spiking model (right). Each unit was classified as displaying one of the pattern types in its average PSTH histogram (across 20 trials). The ordinate indicates the percentage of trials in which the dominant pattern (the pattern occurring most often across trials and is typically that which is observed in the averaged PSTH histogram) of the average was actually exhibited by the unit. In the 2-population graph, units 1–100 are all members of one population, while units 101–200 are part of the other population. In the 4-population graph, units 1–50, 51–100, 101–150, and 151–200 are the members of populations 1 through 4 respectively. Note that the overall average variability in pattern expression is the same in the 2- and 4-population models, with the classified pattern type of each unit being displayed in approximately 47% of the trials, and an approximate range of 30–70% of trials showing different patterns on any given trial. However, while the variability is distributed evenly across both populations in the 2-population model, in the 4-population model the variability is significantly greater in units in those populations exhibiting primarily inhibitory patterns (populations 2 and 4).

We next examine the statistics and dynamics of the spiking version of the distributed mean-field models. In the spiking network version of the 2-population mean field model we first replace the populations with two networks of 100 spiking units each, whose activity averaged across units approaches the activity of the populations in the mean field model (Figure 7). We first analyze the range of memory pattern types in the spiking networks during simulated working memory tasks. Average PSTH histograms over 20 simulated trials of a working memory task were generated and examined for each unit in the network (Figure 8). The pattern types and statistics in these units can be directly compared to those occurring in the database of real parietal and prefrontal cells. The results show that within the populations, the range of excitatory and inhibitory patterns occur, in addition to the non-responsive pattern. The average baseline frequencies, delay frequencies and deltas (changes in frequency from baseline to delay period) exhibited by the units for each pattern fall within ranges observed in the real parietal and prefrontal cells (Table 4). Figure 9A (left) shows the distribution of patterns types exhibited by all 200 units of the 2-population spiking network. The most commonly occurring pattern was the non-responsive pattern, followed by the excitatory patterns, and finally the inhibitory patterns. This relative distribution of pattern types is consistent with what is observed in both parietal and prefrontal cell populations (Figures 9B left and right). This distribution also correlates with the areas of the parameter space over which each of the patterns occurred in the phase diagrams of the 2-population model.

**Table 4:** Average firing frequencies (Hz) during the baseline and delay periods, and the frequency difference between baseline and the average of the delay period (delta), for the units exhibiting each of the pattern types by the 2- and 4-population spiking models.

Pattern	1	2	3	4	5	6	7	8	9
Baseline 2-Population Model	5.6	6.5	6.9	3.9	4.9	9.7	3.9	5.2	4.6
Baseline 4-Population Model	7.4	6.9	6.3	11.9	5.5	10.7	5.1	5.1	4.9
Baseline Parietal Neurons	14	16	14	17.5	12	11	17	15.0	10.5
Baseline Prefrontal Neurons	9.5	7.5	8	27	8.0	7.0	9.0	12.0	7.0
Delay 2-Population Model	7.0	8.9	8.7	3.8	5.0	10.2	2.8	2.7	3.1
Delay 4-Population Model	9.4	9.9	7.8	11.5	5.7	10.8	4.6	2.7	3.3
Delay Parietal Neurons	21.5	25	21	18	12.5	11	12.5	7.5	7.0
Delay Prefrontal Neurons	12.5	11.5	10.5	27.5	8.0	7.5	6.5	8.0	5.5
Delta 2-Population Model	1.4	2.4	1.8	−0.1	0.1	0.5	−1.1	−2.5	−1.5
Delta 4-Population Model	2.0	3.0	1.5	−0.4	0.2	0.1	−1.5	−2.4	−1.6
Delta Parietal Neurons	7.5	9.0	7.0	0.5	0.5	0.0	−4.5	−7.5	−3.5
Delta Prefrontal Neurons	3.0	4.0	2.5	0.5	0.0	0.5	−2.5	−4.0	−1.5

Pattern numbers correspond to the following patterns: 1) Response-coupled ramping cell, 2) fixed rate memory cell, 3) cue-coupled (decaying) memory cell, 4) response-coupled ramping cell with no frequency difference between baseline and period D1, 5) Nonresponsive cells, 6) Decaying inhibition cells with no frequency difference between baseline and period D1, 7) decaying inhibited cells, 8) fixed rate delay inhibited cells, and 9) ramping inhibited cells. The approximate averages for each of these pattern types in the real parietal and prefrontal cell database, as determined in [5] are also indicated. Note the frequencies exhibited by the models are similar to those observed in the real cortical cells.

As is the case in real cortical cells, the specific pattern exhibited by a unit in the spiking network in any given trial can vary from the predominant pattern observed in the average PSTH histogram [5]. That is, the pattern that a unit (or real cortical cell) is classified as exhibiting, as determined from the average PSTH pattern, might not be exhibited on some subset of trials. This includes exhibiting different excitatory patterns from trial to trial in delay activated pattern cells, different inhibitory patterns from trial to trial in delay inhibited pattern cells, or even pattern types contrary to the average pattern. For example, the parietal delay activated cells exhibited delay inhibited patterns on 13.2% of the trials, and parietal delay inhibited cells exhibited delay activated patterns on 15.6% of the trials. Prefrontal delay activated cells exhibited delay inhibited firing patterns on 16.1% of the trials, and prefrontal delay inhibited cells exhibited delay activated patterns on 20.1% of the trials. Figure 9C (left) indicates for each unit in the 2-population network the percentage of the total number of simulated trials in which its pattern behavior differed from its dominant pattern type appearing in its average PSTH histogram (different excitatory or inhibitory pattern and/or contrary delay activity). We see that units in the network, on average, exhibit pattern activity different than their classified pattern type on approximately 52.5% of the trials, with that variability being approximately the same in both local networks. Individual units exhibited different patterns over a range from 20% to 70% of the trials. This variability is comparable to that observed in many real neurons during working memory

### Spiking Unit Network: 4-Population Model

We next examine the range of statistic and memory pattern types occurring in the activity of units in a spiking network with four populations of 50 neurons each. In the spiking network we replace the four populations of the mean field model with four networks of 50 spiking units each whose activity averaged across units is the same as the activity of the populations of the mean field model. We first analyze the range of memory pattern types in the spiking networks during simulated working memory tasks. Average PSTH histograms over 20 simulated trials of a working memory task were generated and examined for each unit in the network. As was the case in the 2-network spiking model, the range of excitatory and inhibitory patterns, in addition to the non-responsive pattern are exhibited by the units in the network. The specific baseline frequencies, delay frequencies and deltas (changes in frequency from baseline to delay period) exhibited by the units for each pattern fall within the ranges observed in the real parietal and prefrontal cells (Table 4). Figure 9A (right) shows the distribution of patterns types exhibited by all 200 units of the 4-population spiking network. The relative prominence of different pattern types is similar to that of the 2 population spiking model, with the most commonly occurring pattern being the non-responsive pattern, followed by the excitatory patterns, and finally the inhibitory patterns. Once again this is consistent with the relative percentages of each pattern type observed for the real parietal and prefrontal neurons.

**Figure 10:**
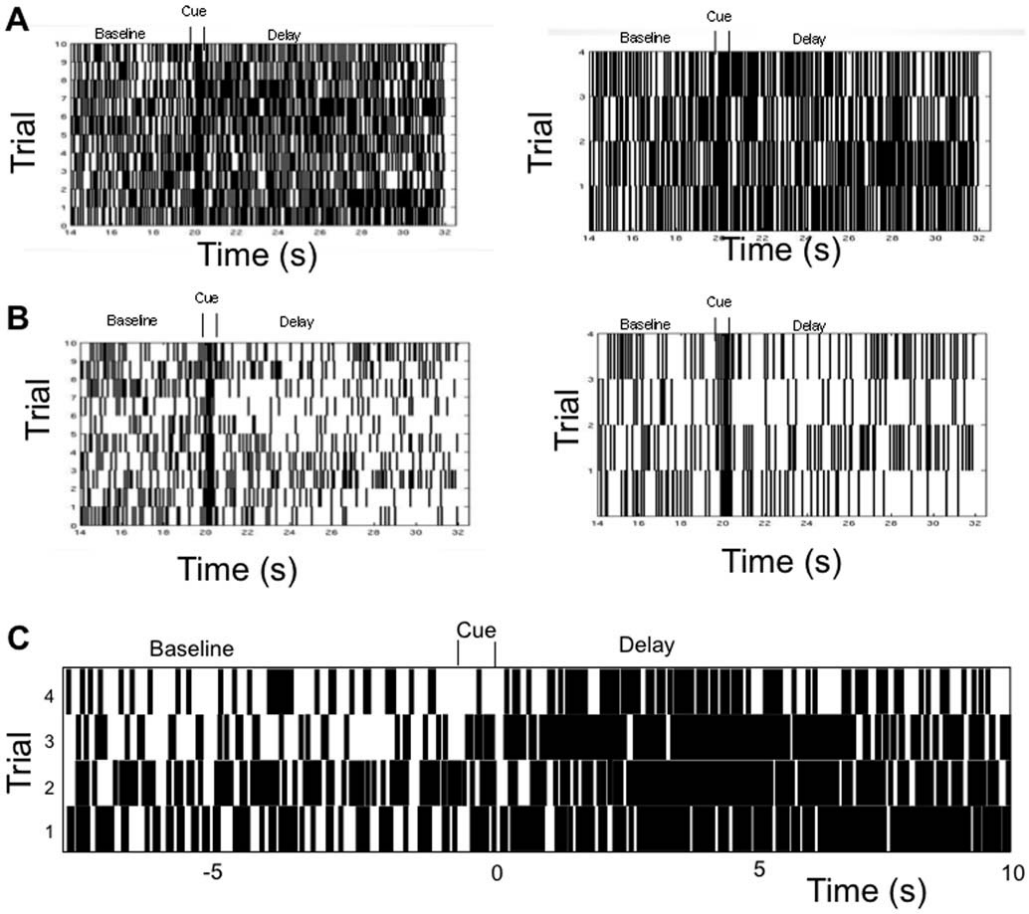
Raster plots of 10 trials (left) for an excitatory (A) and an inhibitory (B) unit from the 4-population 2000 unit spiking network. Note that different pattern types occur on different trials. Figures A and B (right) show an expanded version of 4 of the 10 trials from both units to highlight different patterns exhibited. From top to bottom of the expanded trials, the excitatory unit shows a decaying rate, nonresponsive, increasing rate, and persistent stable firing rate pattern, and the inhibitory unit shows decaying inhibition, stable inhibition, increasing rate excitation, and increasing inhibition. C) Rasters from 4 trials selected from a real neuron recorded from the prefrontal cortex during presentation of the same memorandum of the cross-modal task exhibiting stable persistent activation during the delay in the average PSTH. Although the cells exhibits stable persistent activation on average, on specific trials the cell exhibits the decaying rate activation pattern.

As was the case in the 2-population spiking model, the specific pattern exhibited by a unit in the 4-population spiking network in any given trial of the simulated working memory task can vary from the predominant pattern observed in the average PSTH histogram (Figure 10). Figure 9C (right) indicates for each unit in the network the percentage of the total number of trials in which its pattern behavior differed from its dominant pattern type. We see that units in the network, on average, exhibit pattern activity different than their average classified pattern type approximately 54% of the trials, with individual units exhibiting different patterns over a range of 25% to 70% of trials. This variability is very similar to that observed in the 2-population spiking model, and once again is within the range observed in the real cortical neurons. However, in contrast to the 2-population network, variability is not uniformly distributed across populations. In the 4 population network there is a greater degree of partitioning of the activity of the networks into those primarily exhibiting non-responsive and excitatory patterns, and non-responsive and inhibitory patterns. Populations of units of primarily excitatory or the non-responsive memory pattern types exhibit their predominant pattern much more reliably than those populations of units of primarily inhibitory and non-responsive pattern types. Thus the more distributed architecture resulted in increased reliability in persistently active populations.

### Spiking Unit Network's Variability Scaling With Population Size

**Figure 11:**
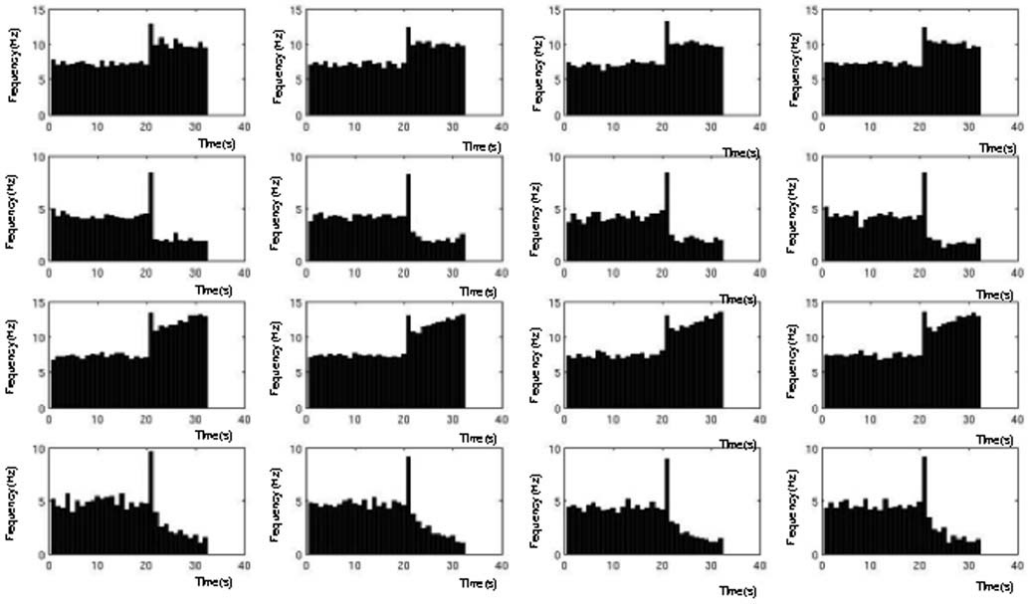
Average PSTH histograms of 16 units randomly chosen from the 2000 unit 4-population spiking model. Note that the entire range of pattern types continue to be exhibited (excitatory and inhibitory stable, decaying, and ramping delays and nonresponsive) with firing statistics similar to real neurons.

**Figure 12:**
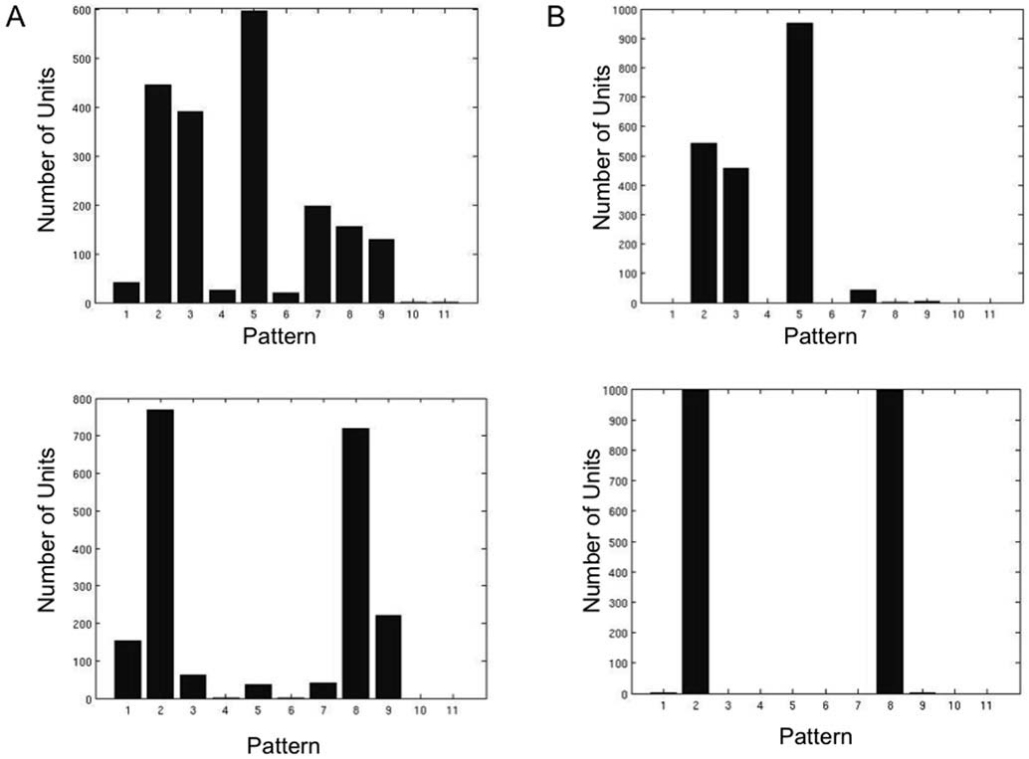
Distributions of patterns exhibited across 20 trials of the simulated task. A) *top*: Distribution of pattern occurrence for the 2-population, 2000-unit spiking network. The distribution is similar to that observed for the 200 unit networks and the parietal and prefrontal neuronal populations. *Bottom*: Distribution of patterns exhibited in the average (across 20 trials) PSTH histograms for the 2-population, 2000-unit spiking network. Note that the distribution of average PSTH exhibits the bistable excitatory and inhibitory patterns more prominently than actually exhibited from trial to trial by the units. B) *top*: Distribution of pattern occurrence for the 4-population, 2000-unit spiking network. The distribution shows similar relative occurrences of the persistent excitatory and inhibitory patterns (stable, ramping, decaying) as all previous networks and real neuronal data. Relative changes in specific pattern occurrences (i.e. decrease in the prevalence of the nonresponsive patter) result from each population's activity approaching that of its corresponding mean firing rate model single pattern with increasing size. Thus a larger number of populations would be needed (i.e. greater than 4) to maintain the relative occurrence of all patterns and thus to maintain an invariant distribution. *Bottom*: Distribution of patterns exhibited in the average (across 20 trials) PSTH histograms for the 4-population, 2000-unit spiking network. Note that the average PSTHs exhibited are essentially all the bistable excitatory and inhibitory patterns. Thus analysis of average patterns across trials might falsely indicate that these are the only patterns of relevance occurring.

We next examine the dependence of pattern type, firing rate statistics and variability as a function of population size. To do this we produced a 2- and 4-population spiking model as above consisting of 2000 units. We first analyze the range of memory pattern types in the spiking networks during simulated working memory tasks. Average PSTH histograms over 20 simulated trials of a working memory task were generated and examined for each unit in the network. As was the case in the 2- and 4 population spiking networks consisting of 200 units, the range of excitatory and inhibitory patterns, in addition to the non-responsive pattern are exhibited by the units in the network (Figure 11). The specific baseline frequencies, delay frequencies and deltas (changes in frequency from baseline to delay period) exhibited by the units for each pattern fall within the ranges observed in the real parietal and prefrontal cells. Figure 12 shows the distribution of patterns types exhibited by all 2000 units of the 2- and 4-population spiking networks. The relative percentage of excitatory and inhibitory patterns is similar to that observed in the 200 unit networks with excitatory patterns being slightly more prominent than inhibitory patterns.

The firing rate model produces a trajectory in the phase plane which corresponds to a specific pattern type. Depending on the connections and other parameters, the stimulus causes the trajectory to remain above or below the separatrix of the phase space. In terms of the spiking model the firing rate model trajectory corresponds to the mean of the trajectory of all units. Depending on how close to the separatrix that mean trajectory is after the stimulus, fluctuations about the mean from various sources of stochasticity in the spiking model will result in a probability that units will make transitions to trajectories corresponding to pattern types different than that of the mean trajectory. The resulting pattern types will have a distribution reflecting this. Conversely the closer the system is to one of the stable attractors of the system, the less probable it is for a given level of noise that the system trajectory will depart from the pattern of the mean trajectory.

**Figure 13:**
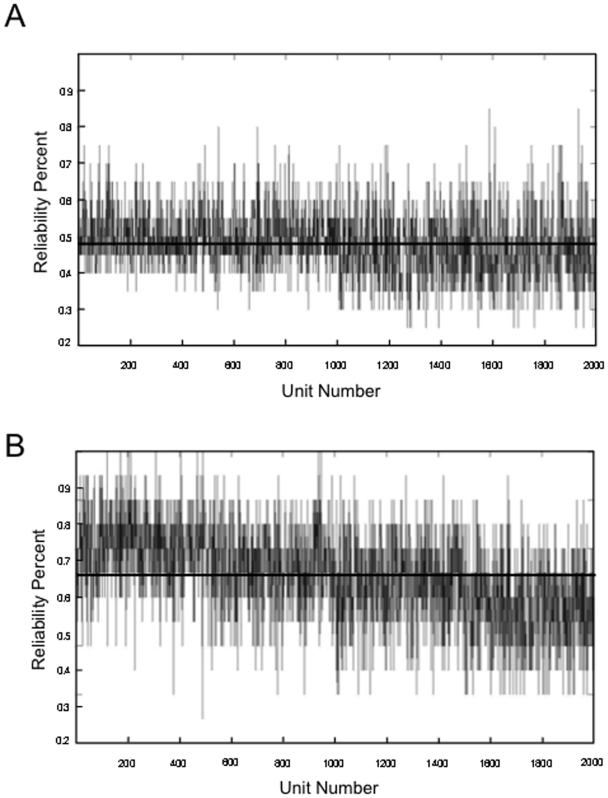
Reliability of units in the 2000 unit networks for exhibiting from trial to trial the pattern indicated by each unit's average PSTH histogram. A) Reliability of each unit in the 2-population, 2000 unit network. The average reliability (percentage of trials exhibiting the pattern observed in the average PSTH histogram) ranges from approximately 40–100% of trials, with an average of approximately 47%. This is similar to that observed for the 200 unit 2- and 4- population spiking models. B) Reliability of each unit in the 4-population, 2000 unit network. The average reliability is significantly higher across all 2000 units (approximately 75%) although the range of variability of individual units is similar to that observed in each of the previous networks. These reliability values are similar to that observed in the real cortical data of parietal and prefrontal cells.

There are 3 primary sources of stochasticity in the spiking model networks, not present in the mean field model that produce fluctuations resulting in units behavior departing from the single pattern type of the mean trajectory: 1) heterogeneity in the connections between units, 2) heterogeneity in the maximum facilitation, and 3) the noise present in all the units' activity. Increasing population size reduces the source of noise resulting from heterogeneous connections, and thus reduces the overall amplitude of fluctuations. Figure 13 shows the reliability with which units in the 2000 unit 2- and 4-population spiking models exhibit there dominant patterns. It can be seen that neurons exhibit their dominant pattern more reliably than in the 200 unit network. However, increasing population size cannot eliminate type variability across trials particularly when the system is near the separatrix. It can be seen from Figure 13 that the average reliability of units expressing a single pattern type across the overall network is greater for the 2000 unit networks than the 200 unit networks (75% vs. 47% respectively in the 4 population model). However, individual units still exhibit high variability in the pattern type exhibited from trial to trial ranging between exhibiting the dominant pattern type 100% of the time to approximately as low as 45% of trials. Thus while reliability in firing can be achieved by increasing population size and averaging across units of a population, this does not eliminate transitions by units from the mean pattern type or from their dominant pattern type from trial to trial.

A reduction in the source of variability due to noise present in all neurons during simulation—i.e. the Weiner noise–can be achieved by averaging across trials. Figure 12A and 12B (bottom) shows the distribution of the average histograms obtained for the units in the 200 and 2000 unit population models across 20 trials. It can be seen that this averaging produces a distributions which primarily consist of patterns corresponding to the canonical bistable persistent activity (activation and inhibition). While this type of averaging is not physiologically relevant in the sense that populations carry out working memory each trial, and not as an average across trials, it does represent the typical averaging carried out to characterize cell behavior in studies of working memory (i.e. average PSTH histograms across trials determine cell pattern type).

**Figure 14:**
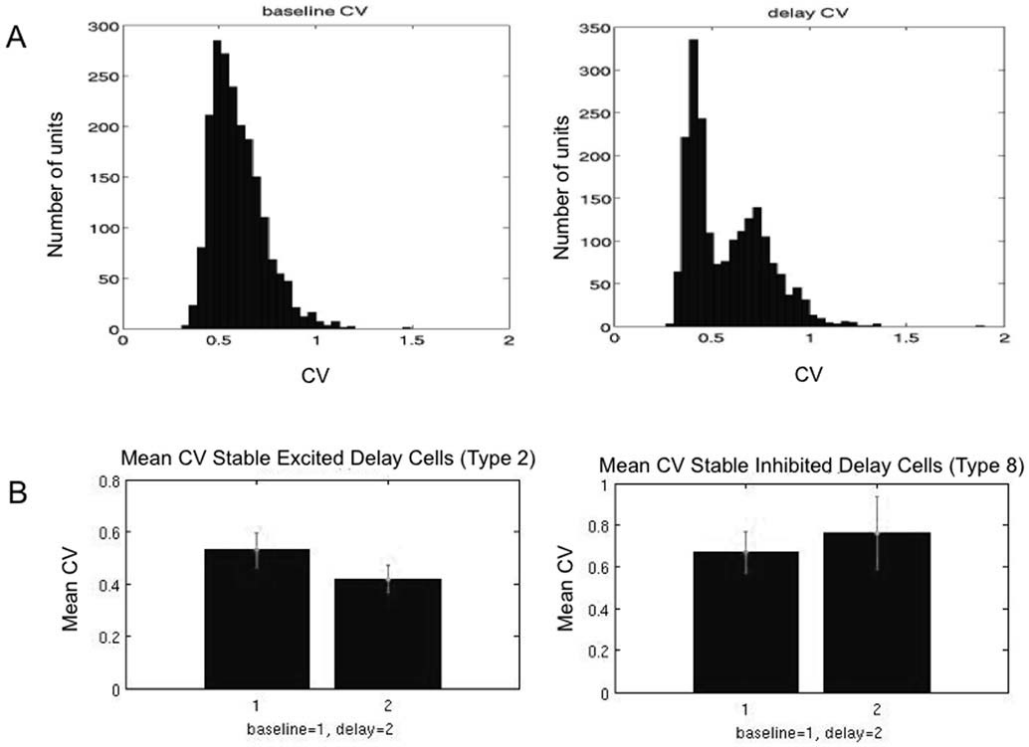
Analysis of spiking variability. A)Distributions of the coefficient of variation of the interspike intervals for units of the 2000 unit spiking model during the baseline and delay. Distributions correspond to cells exhibiting all patterned delay behaviors. High variability in ISI firing occurs during baseline and delay periods. Delay CVs show a bimodal distribution across all pattern types. B) Mean baseline and delay ISI CV for stable persistent excitation delay cells (left) and stable persistent inhibition delay cells (right). The coefficient of variation decreases from baseline to delay in excited delay cells and increases in inhibited delay cells in agreement with the behavior observed in the database of prefrontal and parietal cells.

An analysis of the intra-trial variance of firing rate in the model units revealed high variability in the distribution of ISIs during both baseline and delay periods of the model (Figure 14a). The CV of ISIs in the majority of units ranged in the baseline across all pattern types between 0.4 and 1 with a mode of approximately 0.6. During the delay period the distribution of intra-trial ISI CVs was bimodal with peaks at approximately 0.45 and 0.75 and most units falling within the range of 0.4 and 1 as in the baseline. These ranges of the CV overlap significantly with that observed in the real prefrontal and parietal cell populations, although their overall means are lower. Focusing on the stable excitation and inhibitory patterned activity, units exhibited decreasing average CV from baseline to the delay period in stable excitatory pattern units, and increasing average CV from baseline to the delay period in stable inhibitory pattern units (Figure 14b). In the real parietal and prefrontal cell populations, stable excitatory and inhibitory cells exhibit high CV in their ISIs during both baseline and delay, with the CV decreasing from baseline to delay in stable excitation cells and increasing in stable inhibitory cells. In parietal cortex, the CV of the ISIs in cells exhibiting stable persistent excitation significantly decreased (p<0.001 paired t-test) from and average of 1.17 during the baseline to 1.02 in the delay. In prefrontal cortex the CV in those cells decreased insignificantly from an average of 1.03 to 1.0. In parietal cells exhibiting stable persistent inhibition in parietal cortex, the CV of the ISIs increased insignificantly from 1.19 to 1.2, while in prefrontal cortex the CV increased insignificantly from 1.02 to 1.03 on average.

## Discussion

The results of this study demonstrated that recurrent networks with dynamic synapses inherently produce the different persistent firing rate patterns observed in real cortical neurons during working memory. The persistent patterns produced are robust with respect to variations of the parameters in the network. That is, the different patterns occur over a wide range of values of the parameter space, and given patterns do not occur only for a very narrow set of parameter values. Further, the statistics of those patterns fall within the ranges of variation observed in firing rate pattern behavior of real cortical neurons. For example the changes in firing rate from baseline to the delay period can take values along an apparent continuum with absolute changes in firing rate of less than 100% of the baseline rate. For the majority of persistently activated cells recorded from parietal and prefrontal cortex of primates during working memory this corresponds to changes in firing rate of less than 10 Hz. The present network demonstrates a mechanism beyond previous solution for achieving these realistic low delay firing rates [33]–[35], [46]–[49]. While the expression of any particular delay frequency or rate of ramping or decay of firing rate of the units can be dependent on the particular parameters, the occurrence of any of the working memory patterns takes place across wide continuous ranges of network parameters and inputs, and thus do not involve fine tuning and are stable with respect to noise in the input.

Bistable firing rates are one of the possible activities of the model. However, the present work has focused on the range of working memory-correlated patterns of firing rate and their simultaneous, complementary occurrences in the working memory network as opposed to only fixed states that the networks or their neuronal constituents may adopt. The spiking networks exhibited all of the general patterns correlated with working memory that are observed in the database of microelectrode recordings of parietal and prefrontal cortical neurons. In addition, the statistics and firing rates of the units fall within the ranges observed in real cells, with the occurrence of the different pattern types similar in proportion to that observed in the cortical populations. In terms of the behavior of individual neurons, bistable activity is typically only observed as an average over many trials of a working memory task. Across trials, cells exhibit different average frequencies, and even within individual trials, cells exhibit significant variability in firing rather than a single stable rate [5], [49]–[51]. This is indicated from a high coefficient of variation in both baseline and delay periods in units exhibiting stable delay excitation and inhibition patterns. This is in agreement with database of real parietal and prefrontal stable delay units as well as previous neurophysiological studies in which CVs of within trial ISIs were around 1.0. Changes in CV from baseline to delay period for the model units further agreed with that observed in the parietal and prefrontal database with the CV decreasing for stable excitatory pattern cells, and increasing for stable inhibitory pattern cells. High variability in ISIs has been observed in previous neurophysiological studies [49]–[51], although in some cases the change from baseline to delay observed has been different than that of the present cell populations. This may result from the frequencies or types of persistent patterned activity observed in those studies during the delays (e.g. bursting behavior). In addition to variability within trials of stable persistent activity cells, from trial to trial, neuron activity may adopt specific memory correlated patterns different from the most prominent one that emerges in the average across many trials. This not only includes changing between the different persistent excitatory patterns from trial to trial, but even changing between persistent activation and inhibited patterns. Thus while a population of cells may exhibit a particular pattern with consistency, individual cells of that population do not. In the 2-population spiking model with 200 units variability in firing pattern across trials was the same for both populations, with the majority of units exhibiting changes from their most prominent pattern type (including changing between persistent excitation and inhibited patterns) in 40% to 60% of the trials. In the 4-population spiking model, while the overall variability was essentially the same as in the 2-population model, the variability in pattern across trials depended on the types of patterns prominently exhibited by the particular populations. In populations exhibiting excitatory patterns the majority of units displayed a different pattern on 35% to 45% of the trials, and in populations exhibiting inhibited patterns, the majority of units displayed different patterns on 40% to 60% of the trials. Thus a more distributed architecture resulted in a more reliable occurrence of excitatory memory pattern types within units, in addition to a more stable concomitant occurrence of complimentary pattern types. Working memory therefore appeared to be more stable in the more widely distributed network. The reliability of exhibiting a given pattern across trials increases only partially with the size of the network. Looking at the 4-population network with an order of magnitude more unit results in a network that still exhibits the range of pattern types with relative proportions similar to that seen in the smaller network and in real cortical neurons. Although overall, the average percent of trials that units exhibit their most dominant pattern type increases (i.e. 75% compared to approximately 47%), many units continue to exhibit their dominant pattern on less than a majority of the trials. The reason for this is that increasing population size decreases fluctuations due primarily to stochasticity in the connections between units, which of course does not drop to zero. In addition other sources of stochasticity remain such as noise in unit activity, and stochasticity in the facilitation. Thus given a particular stimulus, units' trajectories in the phase plane will pass with some proximity to the boundary (separatrix) between the fixed bistable states of the system, and given the closeness to the separatrix and the amount of stochasticity, will have a significant chance on any given trial of crossing over to a trajectory corresponding to a different pattern than the mean trajectory of the population, or that which occurs most often for a particular unit. As a result, there is a relatively invariant distribution of pattern occurrence that changes modestly with increasing population size. It is interesting to note that artificially reducing the other sources of stochasticity by for example averaging across trials, that one produces pattern distributions exhibiting essentially only bistable patterns. That is if we look at the average PSTH activity of the networks across trials they tend to be either stable activation cells, or stable inhibition cells. While this type of reduction of stochasticity is not physiologically meaningful since working memory takes place trial to trial and cannot require averaging over many trials, data from unit recording experiments typically report unit activity as average (across trials) PSTH histograms. Thus the prevalence of bistability may be overestimated. Rather in real cortical data as well as in the model we see the variability as in the model.

While the firing pattern varies from trial to trial in cells, there are also significant variations from trial to trial in the delay frequencies for any particular patterns exhibited. The concept of a network with fixed connectivity and bistability between units is not indicated by such activity, and thus a dynamic connectivity is reasonable to consider. The idea of bistable activity corresponding to fixed attractor states may apply at the level of a population of neurons, and could be the essential neuronal correlate of working memory. However, the majority of persistent activity patterns observed consists of cells whose firing rates decay or accelerate during working memory (cue-coupled or response-coupled), and these populations should be taken into account in addition to bistability. In the present model, as stated above, bistable attractor states are present and could correspond to working memory. Here however, a second additional role is suggested for these states in terms of modulating the firing rate activity, resulting in decaying and ramping firing patterns. That is, without necessarily representing memory states in and of themselves, the attractor states allow the network, which represents working memory and its complementary functions, to become active and behave with the necessary dynamics to mediate cross-temporal contingencies. The key component, resulting here from facilitation and observable in the phase plane, is the presence of the bottleneck through which the trajectories of the system pass. The bottleneck modulates the rate at which the trajectories approach the stable attractors, thus creating the patterned activities within the actual range of frequencies observed in the cortex. This mechanism might be present and modulate activity through other components of the network in addition to facilitation. For example Durstewitz [37] has previously demonstrated that a bottleneck or “ghost” of a line attractor could be achieved at a neuronal level through interactions between firing rate output and calcium gated ion channels, generating the climbing firing rate activity of set cells. Therefore, while the bistability (or multiple attractor states) could represent working memory, it plays the additional role of influencing the dynamics of the system such that the resulting trajectories correspond to different classes of working memory behavior (i.e. cue-coupled or response-coupled delay period patterns). The lower attractor state of the system, as is usual, is identified with baseline firing rates. Trajectories of the system not crossing the saddle separatrix remain in the basin of attraction of this attractor and ultimately return to baseline firing rates, adopting one of the classes of persistent activation associated with memory storage. In contrast, trajectories crossing the saddle separatrix approach the stable state corresponding to a higher firing frequency. Depending on the rate at which the system approaches the higher state, the firing pattern adopted is either bistable memory behavior (rapid or very slow approach), or ramping cell behavior associated with preparation for a behavioral or motor response. From these considerations it might be predicted that ramping cells and fixed rate memory cells in working memory should exhibit higher average firing rate changes from baseline than decaying-rate memory cells. An examination of the firing rate changes of prefrontal and parietal cells indicates that this is indeed the case. In parietal cortex, fixed rate memory cells exhibit an average difference between baseline and delay periods of approximately 9 Hz, set cells 7 Hz, and decaying rate memory cells 6 Hz. In prefrontal cortex the same trend is observed with stable rate memory cells exhibiting the greatest mean change in firing rate from baseline to delay period (approximately 4.3 Hz), followed by set cells (3 Hz) and decaying rate memory cells (2 Hz). The fact that the fixed rate memory cells exhibit the largest average change in firing rate from baseline to delay is consistent with some percentage of those cells' activity corresponding to the high firing rate bistable state in addition to those exhibiting only apparent bistability.

The dynamic synaptic facilitation is the component of this model which creates the bottleneck in the phase plane, and gives it its unique characteristics. Specifically it is facilitation which determines the amount of persistent activation, which, since it can adopt a continuous range of values, enables the change in firing rate from baseline to memory period to fall along a continuum. The bottleneck determines the rate at which the firing rates decay towards the baseline attractor (or increases towards the higher firing rate attractor) to adopt the continuum of firing rate values. The decay rate can be sufficiently slow such that no decay or acceleration of firing is observed for the duration of a memory period. Thus the result is an apparent or virtual bistability, which for all intents and purposes can be extended for as long as working memory is defined by the parameters of a working memory task. The fact that the rate at which persistent activation waxes or wanes is highly adjustable is consistent with the behavior of cells in the cortex during working memory. It has been observed in working memory experiments [27], [52]–[53] that the rate of decay and/or the rate of acceleration of persistent activation adjust to the duration of the memory period. The dynamic synapses make this phenomenon easy to incorporate. Adjusting the maximum of facilitation or other parameters, changes the bottleneck so that the rate of decay (or ramping) can become longer or shorter along a continuum.

Another prediction from the dynamics of the model is that the rate of persistent activation correlates with baseline rate. In the majority of delay activated cells, the magnitude of firing rate increases are less than 100% of baseline, with the magnitude of the delay period firing rate change increasing nonmonotonically with baseline rate increases. The largest magnitude increases in delay period frequency are in those cells with the largest baseline firing rates, while the largest percentage changes are those with low baseline rates. This is naturally incorporated in the present model. The range of rates over which the population can exhibit memory cell behavior is bounded by the saddle separatrix. Once facilitation pushes the system's trajectory beyond the separatrix, further increasing facilitation (or judiciously adjusting other parameters) does not result in further continuous increases in persistent activation delay rates, but rather a change in the activation pattern itself. The parameters of the model can be adjusted however, raising the frequency of the baseline state and incrementing the entire range of frequencies within its basin of attraction. Thus both baseline and delay rates increase in a correlated fashion, and due to the nonlinearity of the nullcline of the synaptic activity, the proportional increase in frequency is nonmonotonic.

The specific simultaneous patterns which may be exhibited in the populations are dependent on the relative strength of the inter-population connection strength, the intra-population connection strength, and whether the inter-population connectivities are mutually net inhibitory, or a combination of excitatory and inhibitory. The phase diagram of the 2-population firing rate model reveals a number of behavioral trends. For an excitatory-inhibitory connectivity between populations, the networks can exhibit a range of concomitant activities which includes memory cell activity in both populations, and simultaneous cue-coupled/response-coupled behavior. In contrast, with a mutually inhibitory connectivity between populations these particular behaviors are absent, and simultaneously occurring fixed-rate-memory/response-coupled behavior is present over only an extremely narrow range of the parameters. Thus memory being maintained simultaneously in both cortical areas occurs in the 2-population model only within the E-I connectivity scheme. During working memory, the simultaneous presence of memory cells in prefrontal cortex and another cortical area important to the sensory modality of the memorandum has been indicated by numerous studies. In addition to prefrontal cortex, memory cells have been observed for example in posterior association cortex including inferotemporal cortex [54]–[55], and posterior parietal cortex [56]–[57], and their simultaneous presence in multiple cortical areas have been indicated in imaging studies [55], [58]–[60]. The overlapping presence of cue-coupled and response-coupled cells have also been confirmed and have been implicated in working memory networks [61]. It is suggested that these populations would cooperate and be engaged in the transfer of information from a perceptual network to a motor network. The two populations would cooperate to enable the processing of information from one network to the other with translation from perception into action.

As the network becomes more distributed, increasing to four populations, simultaneous memory cell behavior and cue-coupled/response-coupled behaviors becomes more robust with these concomitant behaviors occurring over a wide continuous range of the parameters as can be observed by the increased areas of those respective behaviors over larger continuous ranges of the parameters in the phase diagrams (Figure 6). While the specific connectivity between populations in different cortical areas in working memory networks is unknown, it is suggestive to consider the possible effects on each population's activity in the model when another is shut down as in reversible lesion studies. For example in the 4-population model, termination of the activity two populations changes the phase diagrams to that of the 2-population model. Depending on the specific local parameters (i.e. the connectivity between populations), the effect can be a net increase in persistent activity, a decrease, or elimination of such activity to a non-responsive pattern. Studies of reversible lesions in which one cortical area is cooled while recording cell activity in another has shown that some cells increase their firing rate during the delay, while other will show decreases or become non-responsive [58], [62]–[63]. A question is whether the net effect of one cortical area on the other is excitatory or inhibitory as might as determined by more cells increasing persistent activation or decreasing it. While no definitive results exist, from these studies the data indicate that more neurons increase their activity in prefrontal cortex as a result of cooling posterior association cortices, while more neurons decrease their activity in posterior association cortex as a result of cooling prefrontal cortex. This could be indicative of an effective E-I coupling between cortical areas. Looking at the potential changes in the phase diagrams of the models going from the 4-population model (with connectivity of both the E-I and I-I type) to 2 populations, such behavior is the general trend over the majority of the parameter space. Further lesion studies or microstimulation studies may elucidate the functional connectivity of global network in light of the model.

In addition to a distributed architecture affecting the stability of memory pattern behavior and modulating activity enabling the occurrence of complimentary patterned behaviors, certain working memory pattern behaviors apparently are exclusively a function of a distributed architecture rather than the facilitation mechanism alone. Particularly the ramping delay inhibition pattern which is observed in the cortical data was present only in the distributed versions of the model. Another phenomenon is the existence of large regions of the parameter space in which one population exhibits the non-responsive pattern, while the other population exhibits memory cell behavior (fixed-rate, decaying, or ramping). In the database of real cells, the majority of neurons from parietal and prefrontal cortex exhibit the non-responsive pattern of behavior. Interspersed within these populations of non-responsive cells are neurons that exhibit the other patterns. From the models we see that the non-responsive pattern is a common part of a working memory network coexisting with the other patterned behaviors. Studies of patterns in spike sequence of such cells [64]–[66] have indicated that while not exhibiting significant differences between baseline and delay firing rates, such cells can exhibit differences in the patterning of the spike sequence in these periods; indicating participation in the working memory network. The fact that the non-responsive pattern arises as a prominent one in the models, overlapping with the range of memory pattern behaviors suggests that these populations may play a role in the dynamics of working memory networks.

It should be noted that the present analysis supplements the general attractor picture rather than replacing, or invalidating it. Cells with apparent bistable activity with high firing rates above baseline, while apparently rare in the cortex [5], may still be a fundamental neuronal substrate of working memory. In the present model not only is this activity present, but also the myriad other patterns with firing statistics and variability similar to those which constitute much of the activity correlated with working memory are accounted for.
